# LRRK2 kinase inhibition reverses G2019S mutation-dependent effects on tau pathology progression

**DOI:** 10.1186/s40035-024-00403-2

**Published:** 2024-03-04

**Authors:** Noah Lubben, Julia K. Brynildsen, Connor M. Webb, Howard L. Li, Cheryl E. G. Leyns, Lakshmi Changolkar, Bin Zhang, Emily S. Meymand, Mia O’Reilly, Zach Madaj, Daniella DeWeerd, Matthew J. Fell, Virginia M. Y. Lee, Dani S. Bassett, Michael X. Henderson

**Affiliations:** 1 Department of Neurodegenerative Science, Van Andel Institute, 333 Bostwick Ave NE, Grand Rapids, MI 49503 USA; 2 Aligning Science Across Parkinson’s (ASAP) Collaborative Research Network, Chevy Chase, MD 20815 USA; 3 Department of Bioengineering, University of Pennsylvania, Philadelphia, PA 19104 USA; 4 Department of Pathology and Laboratory Medicine, Perelman School of Medicine, Institute On Aging and Center for Neurodegenerative Disease Research, University of Pennsylvania, Philadelphia, PA 19104 USA; 5 Neuroscience Discovery, Merck & Co., Inc., Boston, MA 02115 USA; 6 Department of Electrical and Systems Engineering, University of Pennsylvania, Philadelphia, PA 19104 USA; 7 Department of Physics and Astronomy, University of Pennsylvania, Philadelphia, PA 19104 USA; 8 Department of Neurology, University of Pennsylvania, Philadelphia, PA 19104 USA; 9 Department of Psychiatry, University of Pennsylvania, Philadelphia, PA 19104 USA; 10 Santa Fe Institute, Santa Fe, NM 87501 USA; 11 Bioinformatics and Biostatistics Core, Van Andel Institute, 333 Bostwick Ave., NE, Grand Rapids, MI 49503 USA

**Keywords:** G2019S, MLi-2, Cell-to-cell spread, Transmission, Genetic risk, Mapt

## Abstract

**Background:**

Mutations in leucine-rich repeat kinase 2 (*LRRK2*) are the most common cause of familial Parkinson’s disease (PD). These mutations elevate the LRRK2 kinase activity, making LRRK2 kinase inhibitors an attractive therapeutic. LRRK2 kinase activity has been consistently linked to specific cell signaling pathways, mostly related to organelle trafficking and homeostasis, but its relationship to PD pathogenesis has been more difficult to define. *LRRK2*-PD patients consistently present with loss of dopaminergic neurons in the substantia nigra but show variable development of Lewy body or tau tangle pathology. Animal models carrying *LRRK2* mutations do not develop robust PD-related phenotypes spontaneously, hampering the assessment of the efficacy of LRRK2 inhibitors against disease processes. We hypothesized that mutations in *LRRK2* may not be directly related to a single disease pathway, but instead may elevate the susceptibility to multiple disease processes, depending on the disease trigger. To test this hypothesis, we have previously evaluated progression of α-synuclein and tau pathologies following injection of proteopathic seeds. We demonstrated that transgenic mice overexpressing mutant LRRK2 show alterations in the brain-wide progression of pathology, especially at older ages.

**Methods:**

Here, we assess tau pathology progression in relation to long-term LRRK2 kinase inhibition. Wild-type or LRRK2^G2019S^ knock-in mice were injected with tau fibrils and treated with control diet or diet containing LRRK2 kinase inhibitor MLi-2 targeting the IC50 or IC90 of LRRK2 for 3–6 months. Mice were evaluated for tau pathology by brain-wide quantitative pathology in 844 brain regions and subsequent linear diffusion modeling of progression.

**Results:**

Consistent with our previous work, we found systemic alterations in the progression of tau pathology in LRRK2^G2019S^ mice, which were most pronounced at 6 months. Importantly, LRRK2 kinase inhibition reversed these effects in LRRK2^G2019S^ mice, but had minimal effect in wild-type mice, suggesting that LRRK2 kinase inhibition is likely to reverse specific disease processes in G2019S mutation carriers. Additional work may be necessary to determine the potential effect in non-carriers.

**Conclusions:**

This work supports a protective role of LRRK2 kinase inhibition in G2019S carriers and provides a rational workflow for systematic evaluation of brain-wide phenotypes in therapeutic development.

**Supplementary Information:**

The online version contains supplementary material available at 10.1186/s40035-024-00403-2.

## Introduction

Parkinson’s disease (PD) is a progressive disease characterized clinically by motor and non-motor symptoms and pathologically by the presence of Lewy bodies and Lewy neurites in the brain [1]. While most patients have no known genetic causes of disease, there are rare familial cases in which a genetic cause has been identified. Mutations in the leucine-rich repeat kinase 2 gene (*LRRK2*) are the most common cause of familial PD [2]. LRRK2 is a large protein with both kinase and GTPase domains and has been reported to participate in a number of cellular functions, from lysosomal homeostasis to vesicular trafficking [3]. Most identified familial mutations, including the most common p.G2019S [4], lead to elevated LRRK2 kinase activity [2]. For these reasons, LRRK2 kinase inhibitors have been pursued for the past 15 years as potential therapeutics for individuals with *LRRK2* mutations [5]. One recent report has also suggested that the LRRK2 kinase activity may be elevated in idiopathic PD patients [6], extending the potential utility of LRRK2 kinase inhibitors to this much larger population. However, despite years of research, the connection between LRRK2 kinase activity and PD pathophysiology remains unclear. Therefore, while several LRRK2 kinase inhibitors have shown excellent target engagement, it is uncertain what effect LRRK2 kinase inhibition may have on PD pathology or progression.

PD is characterized neuropathologically by the loss of dopaminergic neurons in the substantia nigra and the accumulation of α-synuclein aggregates in the form of Lewy bodies and Lewy neurites [7]. As disease progresses, up to 90% of PD patients also accumulate tau pathology, and tau pathology is associated with more extensive Lewy pathology and a worse prognosis for patients [8]. Since the identification of *LRRK2* mutations as a cause of familial PD, the pathology in *LRRK2*-PD has been less clear. The loss of substantia nigra neurons is ubiquitous in *LRRK2*-PD cases, but 21%–54% of *LRRK2* mutation carriers lack α-synuclein Lewy pathology [9–11]. Tau pathology is also common in *LRRK2*-PD cases, ranging from 79% to 100% [11, 12], similar to the incidence of tau pathology in idiopathic PD, leading to the hypothesis that *LRRK2* mutations could predispose patients to the accumulation of either α-synuclein or tau pathology. While multiple studies have suggested that *LRRK2* mutations are not independently associated with primary tauopathies such as progressive supranuclear palsy or corticobasal degeneration [13, 14], Alzheimer’s disease-type tau pathology still appears consistently with *LRRK2*- and idiopathic PD [11].

Recent work from several groups has therefore sought to determine the impact of LRRK2 kinase activity on α-synuclein and tau pathologies in animal models to provide insight into a relevant clinical outcome for LRRK2 kinase inhibition in clinical trials. Studies in mouse models of α-synucleinopathy have found that LRRK2^G2019S^ transgenic mice have similar overall pathology to wild-type mice, but with enhanced pathology in specific regions [15–17]. Similarly, in mouse models of tauopathy, LRRK2^G2019S^ appears to enhance the progression of tau pathology [18, 19]. These studies have identified changes in pathology patterns that may be related to the LRRK2 kinase activity and would therefore be amenable to reversal with LRRK2 kinase inhibition. However, a caveat of each of these studies was the use of transgenic overexpression of mutant LRRK2.

We therefore sought to assess the impact of LRRK2 kinase inhibition on tau pathology using a validated seed-based progressive tauopathy mouse model. Wild-type and LRRK2^G2019S^ knock-in (KI) mice were injected with Alzheimer’s disease (AD)-derived tau and concurrently treated with control diet or LRRK2 kinase inhibitor MLi-2 in diet targeting the IC50 or IC90 of LRRK2 based on previous research [20]. The mice tolerated chronic LRRK2 kinase inhibition well over 3–6 months of dosing and MLi-2 was estimated to achieve 50%–100% inhibition of LRRK2 kinase activity at medium and high doses, respectively. We then examined tau pathology systematically in 844 regions of the brain in all mice. LRRK2^G2019S^ mice showed a time-dependent enhancement of tau pathology, particularly in cortical brain regions, consistent with previous studies in transgenic mice. Remarkably, this alteration appeared kinase-dependent, since LRRK2 kinase inhibition was able to reverse this effect. We also found that LRRK2 kinase inhibition was not protective in wild-type mice. To gain further insight into the mechanism underlying these brain-wide changes, we computationally modeled pathology progression based on linear diffusion through the anatomical connectome. We found that diffusion through anatomical connectivity was a reliable predictor of pathology progression in all mice. LRRK2^G2019S^ mice had a reversal in spread direction induced by LRRK2 kinase inhibition. Together, the findings of this study demonstrate that LRRK2^G2019S^ has network-level effects on tau pathology progression that are reversible with LRRK2 kinase inhibition. Further, broad assessments of pathology and network modeling provide more systematic assessment of complex phenotypes in neurodegenerative disease models than more common targeted approaches.

## Materials and methods

### Mice

All housing, breeding, and procedures were performed according to the NIH Guide for the Care and Use of Experimental Animals and approved by the University of Pennsylvania’s Institutional Animal Care and Use Committee. LRRK2^G2019S^ knock-in mice (C57BL/6-*Lrrk2*^*tm4.1Arte*^, RRID: IMSR_TAC:13,940) have been previously described [21]. Mice were bred as heterozygotes, and homozygous (+ / + or G2019S/G2019S) littermates were used for experiments. Both male (*n* = 30) and female (*n* = 35) mice were used. They were 3–4 months old at the time of injection.

### MLi-2 compound administration

MLi-2 was synthesized at Merck & Co., Inc. (Boston, MA). Diet was prepared at Research Diets (New Brunswick, NJ), and formulated to deliver a dose of 0, 10, or 60 mg/kg MLi-2 per day, based on an average daily food consumption of 4 g per 30 g mouse. The base for each diet was the vehicle diet (D01060501; Research Diets).

### Conduritol-β-epoxide (CBE) administration

Archival tissues from four mice treated with the glucocerebrosidase inhibitor CBE in a previous study [22] were used in this study. The slides were stained using standard immunofluorescence as described below.

### Human tissue

All procedures were done in accordance with local institutional review board guidelines of the University of Pennsylvania. Written informed consent for autopsy and analysis of tissue sample data was obtained either from patients or their next-of-kins. All cases used for extraction of paired helical filament (PHF) tau (Additional file 1: Table S1) were Braak stage VI and were selected based upon a high burden of tau pathology by immunohistochemical staining. The cases used for extraction were balanced by sex (female = 2; male = 1) and collected an average of 14 h post-mortem. After extracting all three cases and confirming the seeding capacity of tau, cases were pooled for injection and all mice were injected with the same AD PHF pool.

### Human brain sequential detergent fractionation for PHF tau extraction

Frozen postmortem human frontal or temporal cortex tissues containing abundant tau-positive inclusions were selected for sequential extraction after immunohistochemical examination of these samples as described [23] using previously established methods. These brain tissues were sequentially extracted with increasing detergent strength as previously described [24], and a published protocol is available (10.17504/protocols.io.q26g7p9y8gwz/v1). After thawing, meninges were removed and gray matter was carefully separated from white matter. Gray matter was weighed and suspended in nine volumes (*w*/*v*) of high-salt (HS) buffer (10 mM Tris–HCL (pH 7.4), 800 mM NaCl, 1 mM EDTA, 2 mM dithiothreitol (DTT), protease and phosphatase inhibitors and PMSF) with 0.1% sarkosyl and 10% sucrose, followed by homogenization with a dounce homogenizer and centrifugation at 10,000×*g* for 10 min at 4 °C. The resulting pellet was re-extracted with the same buffer conditions and the supernatants from all extractions were filtered and pooled.

Additional sarkosyl was added to the pooled supernatant to reach a final concentration of 1% and the supernatant was rocked for 1 h at room temperature. The samples were then centrifuged at 300,000×*g* for 60 min at 4 °C. The pellet, which contains pathological tau, was washed once with PBS and resuspended in 100 μl of PBS per gram of gray matter followed by passing through a 27G/0.5 inch needle. The pellets were further suspended by brief sonication (QSonica Microson™ XL-2000; 20 pulses; setting 2; 0.5 s/pulse). The suspension was centrifuged at 100,000×*g* for 30 min at 4 °C. The pellet was suspended in one-fifth to one-half of the pre-centrifugation volume, sonicated briefly (60–120 pulses; setting 2; 0.5 s/pulse) and centrifuged at 10,000×*g* for 30 min at 4 °C. The final supernatant was utilized for all studies and is referred to as AD PHF tau. All extractions were characterized by Western blotting for tau, sandwich ELISA for α-synuclein and Aβ_1–40_ and Aβ_1–42_, and validated by immunocytochemistry in primary neurons from non-transgenic mice. For the extractions used in this study, tau constituted 9%–11% of the total protein, while α-synuclein and Aβ constituted 0.008% or less of the total protein.

### Sandwich ELISA

Characterization of α-synuclein, Aβ_1–40_, and Aβ_1–42_ from AD PHF preparations by sandwich ELISA has been described previously [24], and a published protocol is available (10.17504/protocols.io.261ged83ov47/v1). Assays were performed on 384-well MaxiSorp clear plates (ThermoFisher, Waltham, MA). The plates were coated with well-characterized capture antibodies (α-synuclein: Syn9027 (Center for Neurodegenerative Disease Research, Philadelphia, PA); Aβ_1–40_ and Aβ_1–42_: Ban50 (Fujifilm Wako, Richmond, VA, Cat# 013-26873, RRID: AB_3076195)) at 4 °C overnight, washed, and blocked with Block Ace (AbD Serotec, Raleigh, NC) overnight at 4 °C. The AD PHF preparations were diluted at 1:100 and added to plates. Meanwhile, serial dilutions of recombinant α-synuclein, recombinant T40, or peptides for monomeric Aβ_1-40_ and Aβ_1-42_ were used as the standards. The plates were incubated overnight at 4 °C, then washed and incubated with reporter antibodies (tau: BT2 (Thermo Fisher Scientific, Cat# MN1010, RRID: AB_10975238) + HT7 (Thermo Fisher Scientific, Cat# MN1000, RRID: AB_2314654); α-synuclein: MJF-R1 (Abcam, Cat# ab138501, RRID: AB_2537217); Aβ_1–40_: BA27 (Fujifilm Wako, Cat# 014-26923, RRID: AB_3076198); Aβ_1–42_: BC05 (Fujifilm Wako, Cat# 010-26903, RRID: AB_3076199) overnight at 4 °C. Plates were washed and incubated with HRP-conjugated secondary antibodies for 1 h at 37 °C. Color was developed with a 1-Step Ultra TMB-ELISA Substrate Solution (Thermo Fisher Scientific) for 10–15 min. The reaction was quenched with 10% phosphoric acid and the plate was read at 450 nm on a plate reader (M5, SpectraMax, Molecular Devices, San Jose, CA).

### Stereotaxic injection

All surgery experiments were performed in accordance with the protocols approved by the Institutional Animal Care and Use Committee (IACUC) of the University of Pennsylvania. AD PHF tau from individual extractions was vortexed and diluted with DPBS to 0.4 mg/ml. Tau was sonicated in a cooled bath sonicator at 9 °C (Diagenode Bioruptor®; 20 cycles; setting medium; 30 s on, 30 s off). Mice were injected when 3–4 months old. The mice were deeply anaesthetized with ketamine/ xylazine/ acepromazine and injected unilaterally by insertion of a single needle into the right forebrain (coordinates: -2.5 mm relative to Bregma, + 2.0 mm from midline) targeting the hippocampus (2.4 mm beneath the skull) with 1 µg tau (2.5 µl). The needle was then retracted to 1.4 mm beneath the skull, targeting the overlaying cortex and another 1 µg tau (2.5 µl) was injected. The needle was left in place for 2 min following the injection. Injections were performed using a 25-µl syringe (Hamilton, NV) at a rate of 0.4 µl/min. After 3–6 months, the mice were perfused transcardially with PBS, and brains were removed and underwent overnight fixation in 70% ethanol in 150 mM NaCl, pH 7.4. After perfusion and fixation, the brains were processed into paraffin via sequential dehydration and perfusion with paraffin under vacuum (70% ethanol for 2 h, 80% ethanol for 1 h, 95% ethanol for 1 h, 95% ethanol for 2 h, 3 times 100% ethanol for 2 h, xylene for 30 min, xylene for 1 h, xylene for 1.5 h, 3 times paraffin for 1 h at 60 °C). Brains were then embedded in paraffin blocks, cut into 6-µm sections and mounted on glass slides. Slides were then stained using standard immunohistochemistry as described below.

### Hematoxylin and eosin staining

Mice were perfused transcardially with PBS. Lung and kidney tissues were collected and post-fixed in 10% neutral-buffered formalin (NBF). The lung tissues were first inflated by injection with 10% NBF. A published protocol for staining is available (10.17504/protocols.io.kxygx3q9zg8j/v1). After perfusion and fixation, both types of tissues were processed into paraffin via sequential dehydration and perfusion with paraffin under vacuum (70% ethanol for 2 h, 80% ethanol for 1 h, 95% ethanol for 1 h, 95% ethanol for 2 h, 3 times 100% ethanol for 2 h, xylene for 30 min, xylene for 1 h, xylene for 1.5 h, 3 times paraffin for 1 h at 60 °C). Following fixation and paraffin processing, both lung and kidney tissues were stained with hematoxylin and eosin dyes for morphological examination. Sections were immersed in Harris Hematoxylin (Fisher, Cat# 67-650-01) for 1 min, rinsed 2 times in distilled water, differentiated in 0.1% acid alcohol solution for 4 s and rinsed in tap water for 15 min. Sections were then immersed in eosin for 1 min, briefly rinsed in tap water, then dehydrated and mounted with Cytoseal Mounting Media (Fisher, Waltham, MA, Cat# 23-244-256).

### Immunohistochemistry

A published protocol for this method is available (0.17504/protocols.io.5jyl89m9dv2w/v1). Slides were de-paraffinized with 2 sequential 5-min washes in xylenes, followed by 1-min washes in a descending series of ethanols: 100%, 100%, 95%, 80%, 70%. Slides were then incubated in deionized water for one minute prior to microwave antigen retrieval in the BioGenex EZ-Retriever System. Slides were incubated in antigen unmasking solution (Vector Laboratories, Newark, CA; Cat# H-3300) and microwaved for 15 min at 95 °C. Slides were allowed to cool for 20 min at room temperature and washed in running tap water for 10 min. Slides were incubated in 7.5% hydrogen peroxide in water to quench endogenous peroxidase activity, washed for 10 min in running tap water and 5 min in 0.1 M Tris (diluted from 0.5 M Tris made from Tris base and concentrated hydrochloric acid to pH 7.6), and then blocked in 0.1 M Tris/2% fetal bovine serum (FBS) for 15 min or more. Slides were incubated with primary antibody in 0.1 M Tris/2% FBS in a humidified chamber overnight at 4 °C.

To stain pneumocytes, the slides with microwave antigen retrieval as described above, were incubated with anti-prosurfactant protein C antibody (proSP-C, Millipore Sigma, Cat# AB3786, RRID: AB_91588) at 1:4000. After washing with 0.1 M Tris for 5 min, the slides were incubated with biotinylated goat anti-rabbit (Vector, Cat# BA1000, RRID: AB_2313606) or horse anti-mouse (Vector, Cat# BA2000, RRID: AB_2313581) IgG in 0.1 M tris/2% FBS (1:1000) for 1 h. The slides were washed with 0.1 M Tris for 5 min, then incubated with avidin–biotin solution (Vector, Cat# PK-6100, RRID: AB_2336819) for 1 h, and washed for 5 min with 0.1 M Tris. Color was developed with ImmPACT DAB peroxidase substrate (Vector, Cat# SK-4105, RRID: AB_2336520) and counterstained briefly with Harris Hematoxylin (Fisher, Cat# 67-650-01). The lung ProsP-C data were grouped by object area (μm^2^) and plotted versus relative frequencies.

For phosphorylated tau, pS202/T205 tau (AT8, ThermoFisher, Cat# MN1020, RRID: AB_223647) was used at 1:10,000. The primary antibody was rinsed off with 0.1 M Tris and slides were incubated in 0.1 M Tris for 5 min. Primary antibody was detected using the BioGenex Polymer detection kit (Cat# QD440-XAK) according to the manufacturer’s protocol as outlined below. The slides were incubated in 50% Enhancer solution in 0.1 M Tris/2% FBS for 20 min, washed with 0.1 M Tris, incubated in 0.1 M Tris for 5 min, and incubated in 0.1 M Tris/2% FBS for 5 min. Slides were then incubated with 50% Poly-HRP in 0.1 M Tris/2% FBS for 30 min. The Poly-HRP was rinsed off with 0.1 M Tris, and slides were then incubated for 5 min with 0.1 M Tris. Color was developed with ImmPACT DAB peroxidase substrate (Vector, Cat# SK-4105) for 10 min. DAB was rinsed off with 0.1 M Tris, the slides were incubated in distilled water for 5 min and then counterstained briefly with Harris hematoxylin (ThermoFisher, Cat# 6765001).

The slides were washed in running tap water for 5 min, dehydrated in ascending ethanol for 1 min each (70%, 80%, 95%, 100%, 100%), then washed twice in xylenes for 5 min and coverslipped in Cytoseal Mounting Media (Fisher, Cat# 23-244-256). The slides were scanned on an Aperio AT2 microscope using a 20 × objective (0.75 NA) into ScanScope virtual slide (.svs) files. Digitized slides were then used for quantitative pathology.

### Immunofluorescence

A published protocol for this method is available (0.17504/protocols.io.5jyl89m9dv2w/v1). Slides were de-paraffinized with 2 sequential 5-min washes in xylenes, followed by 1-min washes in a descending series of ethanols: 100%, 100%, 95%, 80%, 70%. The slides were then incubated in deionized water for one minute prior to microwave antigen retrieval in the BioGenex EZ-Retriever System. Then the slides were incubated in antigen unmasking solution (Vector Laboratories; Cat# H-3300) and microwaved for 15 min at 95 °C. The slides were allowed to cool for 20 min at room temperature, washed for 5 min in 0.1 M Tris, and blocked in 0.1 M Tris/2% FBS for 15 min or more. Then the slides were incubated with primary antibody in 0.1 M Tris/2% FBS in a humidified chamber overnight at 4 °C. The primary antibodies used were rat anti-glial fibrillary acidic protein (GFAP) (2.2B10, ThermoFisher, Cat# 13-0300, RRID: AB_2532994, 1:1000), rabbit anti-Iba1 (Wako, Cat# 019-19741, RRID: AB_839504, 1:500), and pS202/T205 tau (AT8, ThermoFisher, Cat# MN1020, RRID: AB_223647, 1:1000). Primary antibodies were rinsed off with 0.1 M Tris, and the slides were incubated in 0.1 M Tris for 5 min, in 0.1 M Tris/2% FBS for 5 min, then with fluorescently-labeled secondary antibodies diluted in 0.1 M Tris/2% FBS for 3 h. Finally, the slides were washed 2 × 5 min with 0.1 M Tris and mounted with coverslips in ProLong gold with DAPI (Invitrogen, Cat# P36931). Fluorescent slides were imaged at 20 × magnification on a Zeiss AxioScan 7 microscope.

### Iba-1 fluorescence quantification

To quantify Iba-1-positive cells, annotations of the visual cortex were manually drawn guided by the Allen mouse brain atlas CCFv3. Utilizing the cell classification feature in QuPath, thresholds for control slides and experimental slides were established separately due to the intensity differences that limit the utility of a common cell detection algorithm. The minimum intensity thresholds were established at 2500 and 750, respectively. The cellular detections were then subject to object classification, which successfully differentiated cell bodies from staining artifact and cell processes. A common object classifier was used across control and experimental slides. The sizes and fluorescence intensities of individual cell bodies were averaged across all detections.

### Measurement of pS935 and total LRRK2 in kidney lysates

LRRK2 and pS935 LRRK2 were assessed using the Meso Scale Discovery (MSD) as previously reported [25]. Kidney lysates were prepared in 10 µl/mg MSD lysis buffer (R60TX-2) supplemented with 1 × HALT phosphatase and protease inhibitor (Thermo Fisher, Cat# 78442). Tissue was placed in a 2-ml round bottom tube (Eppendorf) with a steal bead (Biospec Products, 6.35 mm, Cat# 11079635C) and homogenized for 1.5 min at 30% amplitude in a Qiagen TissueLyser at 40 °C. Lysates were centrifuged at 13,000×*g* for 20 min and supernatants were collected for LRRK2 analysis. The total protein level was determined with a Micro BCA Protein Assay according to the kit protocol (Thermo Scientific, Cat# 23235). LRRK2 pS935 and total LRRK2 protein levels were quantified from lysates utilizing respective R-PLEX MSD Antibody Sets (LRRK2pS935, Cat# F211Q-3; total LRRK2, Cat# F211P-3). All incubation steps were performed on a shaker. Streptavidin-coated MSD plates (Cat# L45SA-2) were coated overnight at 4 °C in biotinylated anti-LRRK2pS935 or anti-LRRK2 antibody diluted 1:5 in Diluent 100 (Cat# R50AA-3). Plates were washed three times with 1 × MSD wash buffer (Cat# R61AA-1). The standard curve samples and the supernatants were added to the plate and incubated for 1 h at room temperature. Plates were washed as previously described. SULFO-Tag anti-LRRK2 pS935 (MSD, Cat# F211Q-3) or anti-LRRK2 (MSD, Cat# F211P-3) antibodies were diluted 1:100 in Diluent 100, applied to plate and incubated for 1 h at room temperature. Plates were washed three times, MSD Gold Read Buffer (Cat# R92TG-2) was applied, and plates were immediately read on an MSD plate reader.

### Assessment of plasma levels of MLi-2

Plasma was diluted 1:3 in acetonitrile, vortexed for 2 min, and centrifuged at 4000 RPM for 10 min at 40 °C. Supernatants were collected and assayed for compound levels using liquid chromatography and tandem mass spectrometry analyses as previously described [20].

### Quantitative pathology

Procedures of section selection, registration, and quantification were all done blinded to treatment.

To quantify the size of type II pneumocytes, we implemented an object classifier in the HALO software with a minimum optical density of 0.225. Sizes of individual cells were measured and the overall distribution of sizes was calculated to determine if there was an overall shift in the size of these cells or a shift in the average size per mouse.

To quantify tau pathology and register brain sections to the Allen Brain Atlas CCFv3 (RRID: SCR_017001), we used a modified version of the QUINT workflow [26]. Adaptations are available in a published suite of protocols (10.17504/protocols.io.kqdg3xbkzg25/v1, RRID: SCR_023856). Our workflow utilizes a suite of open access programs for segmentation and spatial registration of histological images of the mouse brain—QuPath [27] (RRID:SCR_018257) for image segmentation, QuickNII [28] and VisuAlign (RRID:SCR_017978) for image registration, Qmask for hemispheric differentiation and Nutil [29] (RRID:SCR_017183) for a combination of segmentation and registration and subsequent quantification. Twelve coronal sections per animal were quantified which contain the majority of regions with pathology. To segment pS202/T205 tau, individual sections were segmented using the pixel classification feature of QuPath. The optical density threshold was set at 0.03 to quantify pathology. All pixels above this threshold were quantified, with obvious artifacts removed. The resulting segmentations were exported at full resolution. Down-sampled brain sections were imported into QuickNII and aligned in 3-dimensional space to the 2017 Allen mouse brain atlas CCFv3. Sections were then imported into VisuAlign where anchor points were generated in the atlas and moved to the corresponding location on the section of interest via non-linear warp transformation. In addition, from the spatial coordinate information derived from QuickNII, the masking tool Qmask was used to generate masks over each brain hemisphere so ipsilateral and contralateral brain regions could be analyzed independently. We then used the quantifier feature in Nutil to align the segmentation from QuPath, the registration from VisuAlign, and the hemispheric masks from Qmask to generate percentage area occupied measures for each brain region. The resulting outputs include pixel quantification with spatial location in the brain mapped to 844 brain regions. The resulting output of each of the 12 coronal sections, was then averaged across brain regions to produce single percent area occupied values for each region.

### Computational models of tau pathology spread

Data were modeled under the assumption that tau proteins spread in both anterograde and retrograde directions along the brain’s structural connectome. Structural data were obtained from the anterograde viral tract-tracing experiments [30]. The high-resolution structural connectome was constructed by estimating the projection strength between each pair of 100-µm-wide voxels within the Allen Institute CCFv3 whole-brain parcellation [31]. Connections were normalized by dividing the edge strength between each source and target region by the size of the source region [31].

The equation used to model bidirectional pathology spread is as follows:

$$\hat y\left( t \right) = {\beta_0} + \;{\beta_a}{\log_{10}}{e^{ - {c_a}{L_a}t}}{x_0} + {\beta_r}{\log_{10}}{e^{ - {c_r}{L_r}t}}{x_0} + \varepsilon \left( t \right)$$

where ${\beta}_{0}$ is an intercept, ${\beta}_{a}$ is a weight for the importance of anterograde spread, ${\beta}_{r}$ is a weight for the importance of retrograde spread, ${c}_{a}$ and ${c}_{r}$ are time constants representing the global speed of anterograde and retrograde spread, respectively, ${L}_{a}$ and ${L}_{r}$ represent the out-degree graph Laplacian for each direction, $t$ is time, ${x}_{0}$ is a vector containing ones in the injection site regions (DG, CA1, CA3, VISam, and RSPagl) and zeros elsewhere, and $\varepsilon$ is an error term[18]. The “optim” function in R was used to identify the combination of anterograde and retrograde time constants that yielded the strongest correlation between actual and predicted pathology for each group.

To compare model parameters across treatment groups, data from mice in each group were resampled 500 times and each resampled dataset was fit using the bidirectional spread model.

### Model validation

We used previously described approaches[18] to evaluate the specificity and performance of our model. To assess the dependence of the model fit on the spatial location of the seed sites, we randomly selected 500 sets of seed regions which had the same average distance from one another as the actual seed sites (1.96 ± 0.196 mm). The bidirectional model described above was then fit for each set of random seed sites, such that ${x_0}$ contained ones in the randomly-selected seed regions and zeros elsewhere.

To evaluate the performance of the bidirectional spread model, we compared fits obtained from models accounting for spread in only one direction (anterograde or retrograde) or based solely on the distance between regions. For each model type, data from mice of both genotypes and treatment groups were pooled and randomly resampled to obtain ${y_{train}}\left( t \right)$ and ${y_{test}}\left( t \right)$ for each time point. The model fit was performed on ${y_{train}}\left( t \right)$ and the fit with ${y_{test}}\left( t \right)$ was then evaluated. This process was repeated 500 times for each model type to obtain distributions of out-of-sample fits.

### Statistical analysis

All primary data are available here: 10.5281/zenodo.10630752. The number of animals analyzed in each experiment, the statistical analysis performed, as well as the* P*-values for all results < 0.05 are reported in the figure legends. In vivo pathological spread data were analyzed and all computations were performed in R (https://www.R-project.org/, RRID: SCR_001905) [32] as described. Type II pneumocyte size was statistically assessed via two distinct methods, the two-way ANOVA (Additional file 1: Fig. S1), and the linear mixed-effects model [33] (Fig. 2) to account for individual mouse variation. A linear mixed-effects model was fit with a random intercept for each mouse and a random coefficient for genotype. Genotype and treatment, as well as their interaction, were included as fixed-effects. Due to the large number of samples per animal, we used the Nelder-Mead optimizer. Area was square-rooted to improve normality of residuals.

For analyses assessing differences in percent area occupied, data were stratified based on brain hemisphere (ipsilateral/contralateral), brain region, MPI, and either genotype or treatment depending on the specific hypothesis test (i.e. stratified on treatment if testing genotype differences and vice-versa). Regions with zero variance across all treatments and genotypes were filtered out. To maintain flexibility and avoid reliance on strong parametric assumptions, robust linear regressions via the MASS package, with the ranked percent area occupied as the outcome and either genotype or treatment as the explanatory variable, were used (https://cran.r-project.org/web/packages/MASS/index.html, RRID: SCR_019125). All models were adjusted for sex and daughter regions when the number of daughter regions was 2 or greater. Effect sizes, including confidence intervals were estimated using the R package *emmeans* (R package version 1.8.3, URL: https://CRAN.R-project.org/package=emmeans, RRID: SCR_018734). Significance was determined using second-generation *P* values based on a null interval of ± 5% difference with 95% confidence intervals [34]. Only second-generation *P* values equal to 0 were considered significant.

## Results

### Chronic LRRK2 inhibition is well-tolerated in wild-type and LRRK2^G2019S^ KI mice

**Fig. 1:**
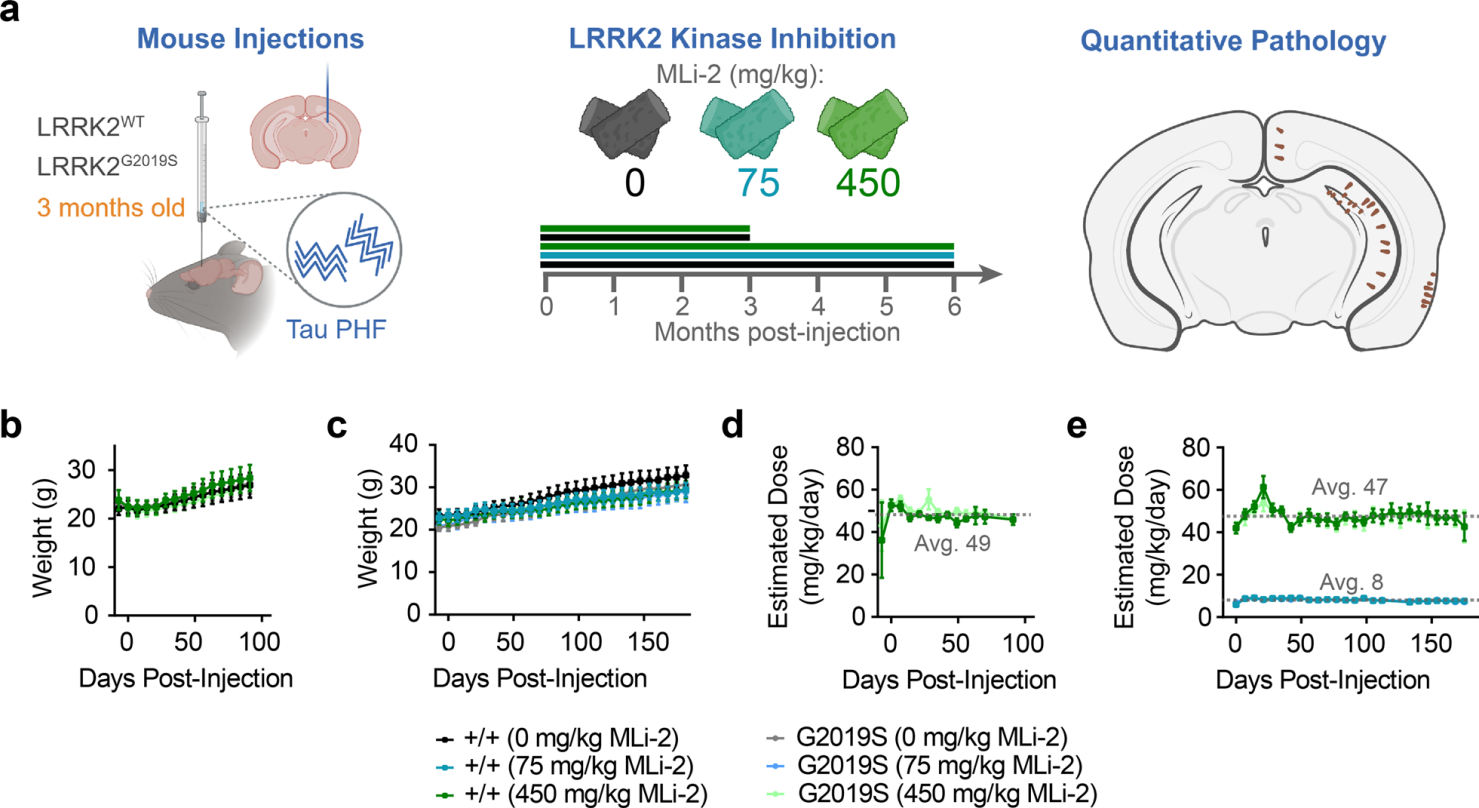
Chronic LRRK2 inhibition is well-tolerated in wild-type and LRRK2^G2019S^ KI mice. **a** LRRK2^G2019S^ KI mice and wild-type littermates at 3 months of age were injected with tau paired helical filaments (PHFs) derived from Alzheimer’s disease brains. At the same time, mice were given access to diet with 0, 75, or 450 mg/kg MLi-2 incorporated. At 3 or 6 months post-injection, mice were sacrificed, and the primary endpoint of brain pathology was assayed. Secondary studies of PK/PD as well as lung and kidney histology were also assayed. **b** Average mouse weights over 3 months of 0 or 450 mg/kg MLi-2 treatment. **c** Average mouse weights over 6 months of 0, 75, or 450 mg/kg MLi-2 treatment. **d** Estimated exposure of mice in the 3-month cohorts to MLi-2 based on diet consumption. **e** Estimated exposure of mice in the 6-month cohorts to MLi-2 based on diet consumption. Mice on the 450 mg/kg diet averaged 48 mg/kg/day exposure to MLi-2, while mice on the 75 mg/kg diet averaged 8 mg/kg/day exposure. Plots display the mean and standard error of each cohort at each time point

We previously used regional pathology quantification and network modeling in wild-type and LRRK2^G2019S^ transgenic overexpression mice to show that seeded tau pathology progresses through the mouse brain in a manner that is constrained by regional connectivity and vulnerability [18]. In LRRK2^G2019S^ transgenic mice, tau pathology is increased in caudal cortical regions and unchanged or decreased in hippocampus and entorhinal cortex [18]. Network modeling of these changes suggested that these changes represented subtle shifts from the anterograde toward the retrograde progression of tau pathology. To determine whether this is also true without overexpression of LRRK2 and whether LRRK2 kinase inhibition can reverse this effect, we injected LRRK2^G2019S^ KI mice [21] and their wild-type littermates with tau PHFs extracted from AD brains (Additional file 1: Table S1)[24] at 3 months of age unilaterally in the hippocampus and overlaying cortex (Fig. 1a). The mice were given ad libitum access to diet formulated to deliver 10 or 60 mg/kg/day of the potent LRRK2 kinase inhibitor MLi-2 [20], or vehicle control diet one week prior to tau injection. Previous work showed that MLi-2 is fully brain penetrant [20] and that these doses achieve approximately IC50 and full inhibition exposures in brain, respectively [35]. Mice were aged to 3 or 6 months post-injection (MPI) and maintained on the altered diet throughout the study. Mice in all groups showed similar weight gain throughout the study (Fig. 1b, c). Mouse diet was weighed weekly, allowing an estimation of diet consumption and expected MLi-2 exposure. Based on these measures, mice fed with the 450 mg/kg MLi-2 diet maintained an exposure of 47–49 mg/kg/day (Fig. 1d, e), while mice fed with the 75 mg/kg MLi-2 diet averaged 8 mg/kg/day exposure (Fig. 1e).

### MLi-2 reduces total and pS935 LRRK2 and enlarges pneumocytes

**Fig. 2:**
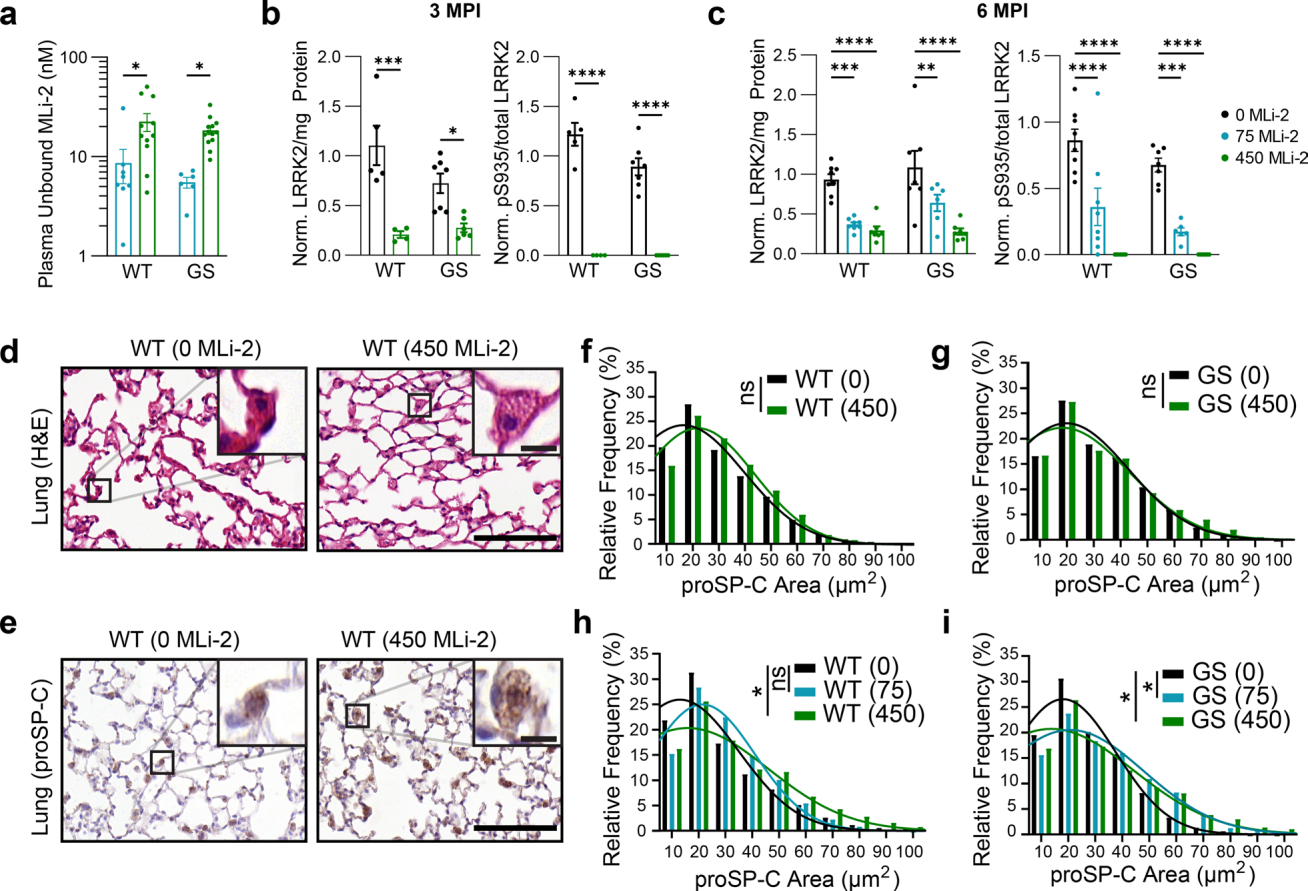
MLi-2 reduces total and pS935 LRRK2 and induces enlargement of pneumocytes. **a** The pharmacokinetic profile of unbound MLi-2 was measured in the plasma of mice following in-diet dosing. *P < 0.05, two-way ANOVA followed by Dunnett’s multiple comparison test to compare doses. **b** Total LRRK2 and pS935 LRRK2 were reduced in the kidney following 3-month treatment with MLi-2. *P < 0.05, ***P < 0.001, ****P < 0.0001, two-way ANOVA followed by Dunnett’s multiple comparison test to compare doses. **c** Total LRRK2 and pS935 LRRK2 were reduced in the kidney following 6-month treatment with MLi-2. ***P < 0.001, ****P < 0.0001, two-way ANOVA followed by Dunnett’s multiple comparison test to compare doses. Values were normalized to the wild-type mice treated with 0 mg/kg MLi-2 across time points. **d** Following hematoxylin and eosin staining, the lungs from mice treated with 450 mg/kg MLi-2 appear to have enlarged type II pneumocytes. Scale bars, 100 µm (main images), 10 µm (insets). **e** Pneumocytes were more easily observed and quantified following staining with an antibody targeting prosurfactant protein C (proSP-C). Scale bars, 100 µm (main images), 10 µm (insets). **f**, **g** proSP-positive pneumocytes were detected and quantified using an automated algorithm. Size distributions of measured signal are plotted. Wild-type and LRRK2^G2019S^ knock-in mice treated for 3 months did not show a significant shift of distribution to larger sizes. ns = not significant, linear mixed effect model. *N* = 40 + cells/mouse from 4 to 7 mice/group. **h**, **i** After 6 months of treatment, wild-type (**h**) and LRRK2^G2019S^ mice (**i**) showed larger distribution of proSP-C-positive pneumocytes at both doses of MLi-2 compared to mice not exposed to MLi-2. *P < 0.05, ns = not significant, linear mixed effect model. *N* = 40 + cells/mouse from 6 to 8 mice/group

To further explore the pharmacodynamic and pharmacokinetic profiles of chronic MLi-2 exposure, plasma, kidneys, and lungs were collected. LC–MS/MS analysis of plasma revealed terminal exposure to 8 nM and 23 nM unbound MLi-2 in mice fed 75 mg/kg and 450 mg/kg diet, respectively (Fig. 2a). Total LRRK2 and pS935 LRRK2 levels were assayed using an established MSD assay in the kidney as readouts of target engagement since the brain was fixed for histology. LRRK2 phosphorylation at S935 is a robust and broadly applicable readout for LRRK2 kinase activity [20, 36, 37]. LRRK2 kinase inhibition significantly reduced LRRK2 levels, though not below the detection limit, whereas pS935 LRRK2 was dramatically reduced by MLi-2 treatment with pS935 LRRK2, becoming undetectable in mice treated with 450 mg/kg MLi-2 (Fig. 2b, c). Overall, the mice on the MLi-2 diet maintained partial to complete inhibition of LRRK2 kinase activity.

Several previous studies have shown that sustained LRRK2 kinase inhibition can lead to morphological phenotypes in the kidney and lung [20, 38–40]. We therefore collected kidney and lung tissues and assayed each for morphological abnormalities. The kidney sections were stained with hematoxylin and eosin and showed no major morphological abnormalities (Additional file 1: Fig. S1a). The lung tissue was stained with hematoxylin and eosin and also immunostained for proSP-C, a marker for pneumocytes. We found that the mice treated with MLi-2, especially those treated with a high dose or for a longer time, showed enlarged pneumocytes (Fig. 2d, e). Quantitative assessment of pneumocyte size based on proSP-C staining revealed that the size distribution of pneumocytes in mice treated with MLi-2 was significantly increased in all groups, except for 3 MPI LRRK2^G2019S^ mice (Fig. 2f–i). We assessed the impact of MLi-2 treatment on individual mice, first with a linear mixed-effects model. Results showed that MLi-2 did not have a significant effect at 3 MPI, but increased pneumocyte size at 450 mg/kg in WT mice (*P* = 0.0498) and at 75 and 450 mg/kg in LRRK2^G2019S^ mice at 6 MPI (*P* = 0.015 and 0.019, respectively). We also found that the average pneumocyte size was increased in LRRK2^G2019S^ mice treated with 75 or 450 mg/kg MLi-2 (Additional file 1: Fig. S1b, c). These results are consistent with previous literature and suggest that a higher dose or more sustained LRRK2 inhibition will result in a stronger pneumocyte phenotype in the lung.

### Quantitative pathology to evaluate the development and spread of tau pathology

To investigate the effect of pharmacological inhibition of LRRK2 on the development and spread of tau pathology, we used an established seed-based model of tauopathy that induces the misfolding of endogenous tau into hyperphosphorylated tau inclusions following injection of AD brain-derived tau into mice [24]. In the present study, we injected LRRK2^G2019S^ knock-in and WT littermates with AD brain-derived PHFs and then aged them to either 3 or 6 months MPI. AD PHF tau induces misfolding of endogenous mouse tau and subsequent progression of phosphorylated tau pathology throughout the mouse brain [18, 24]. Brains were sectioned, and 12 representative sections were selected and stained for phosphorylated tau pathology (AT8, pS202/T205 tau). To quantify the phosphorylated tau pathology throughout the brain, we implemented a segmentation and brain registration platform adapted from the QUINT workflow [26] (Additional file 1: Fig. S2). Segmentation was performed in QuPath using an optical density pixel threshold. In parallel, tissue sections were placed in a three-dimensional atlas using QuickNII [28], and nonlinear warp transformations were performed in VisuAlign. Segmentations were aligned to registered anatomical regions and quantified in the Nutil [29] software. This strategy enabled us to produce highly-reproducible measures of tau pathology burden in 844 anatomical regions across the brain (Additional file 1: Fig. S3).

### LRRK2^G2019S^ causes time-dependent alterations in cortical tau pathology

**Fig. 3:**
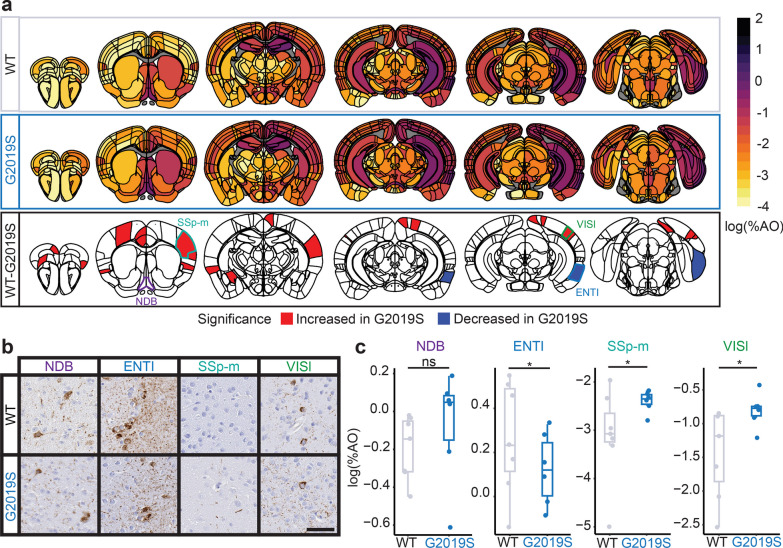
LRRK2^G2019S^ mice show time-dependent alterations in cortical pathology. **a** Anatomic heatmaps of the mean regional tau pathology (AT8, pS202/T205 tau) shown as log (% area occupied) at 6 MPI and second-generation *P*-values, calculated using ranged robust linear regression, of regional statistical significance of wild-type mice compared to G2019S mice (δ*P* = 0). Tau pathology was not quantified in white matter regions, so they are plotted as gray. Analyzed regions shown below are outlined. NDB: diagonal band nucleus; ENTl: entorhinal area, lateral part; SSp-m: primary somatosensory area, mouth; VISl: lateral visual area. **b** Representative images of selected regional tau pathology. Scale bar = 50 μm. **c** Quantification of selected brain regions. Regional tau shown as log (% area occupied). ns = not significant (δ*P* ≠ 0); *: significant (δ*P* = 0). *N* = 7 (WT) and 6 (G2019S)

We have previously demonstrated that LRRK2^G2019S^ overexpressing mice have alterations in tau pathology that are most pronounced at later time points [18]. Since it was not feasible in the current study design to include 9 MPI, we assessed genotype-related differences at 3 and 6 MPI using semi-parametric ordinal regressions while accounting for multiple comparisons. We first assessed tau pathology differences by sex in wild-type mice (Additional file 1: Fig. S4). Interestingly, we found increases in the subcortical pathology at 3 MPI, and less pronounced increases at 6 MPI in male mice. Therefore, all ordinal regression also accounted for sex in estimating differences. We found minimal difference between wild-type and LRRK2^G2019S^ mice at 3 MPI (Additional file 1: Fig. S5, S6). However, by 6 MPI, the pathology was significantly higher in many cortical regions of LRRK2^G2019S^ mice compared with the wild-type littermates, with the exception of entorhinal cortex (Fig. 3a, b). At this time point, pathology had begun to progress past the initial injection sites and their directly connected areas (hippocampus, entorhinal cortex, supramammillary nucleus), and the increase of pathology in the LRRK2^G2019S^ mice was observed in more distally connected sites. Based on previous data in LRRK2^G2019S^ transgenic mice, we would expect the two genotypes to further differentiate at later time points, consistent with a role of LRRK2 in pathology progression, but not in pathology initiation.

### LRRK2 kinase inhibition shows no protective effect in wild-type mice

**Fig. 4:**
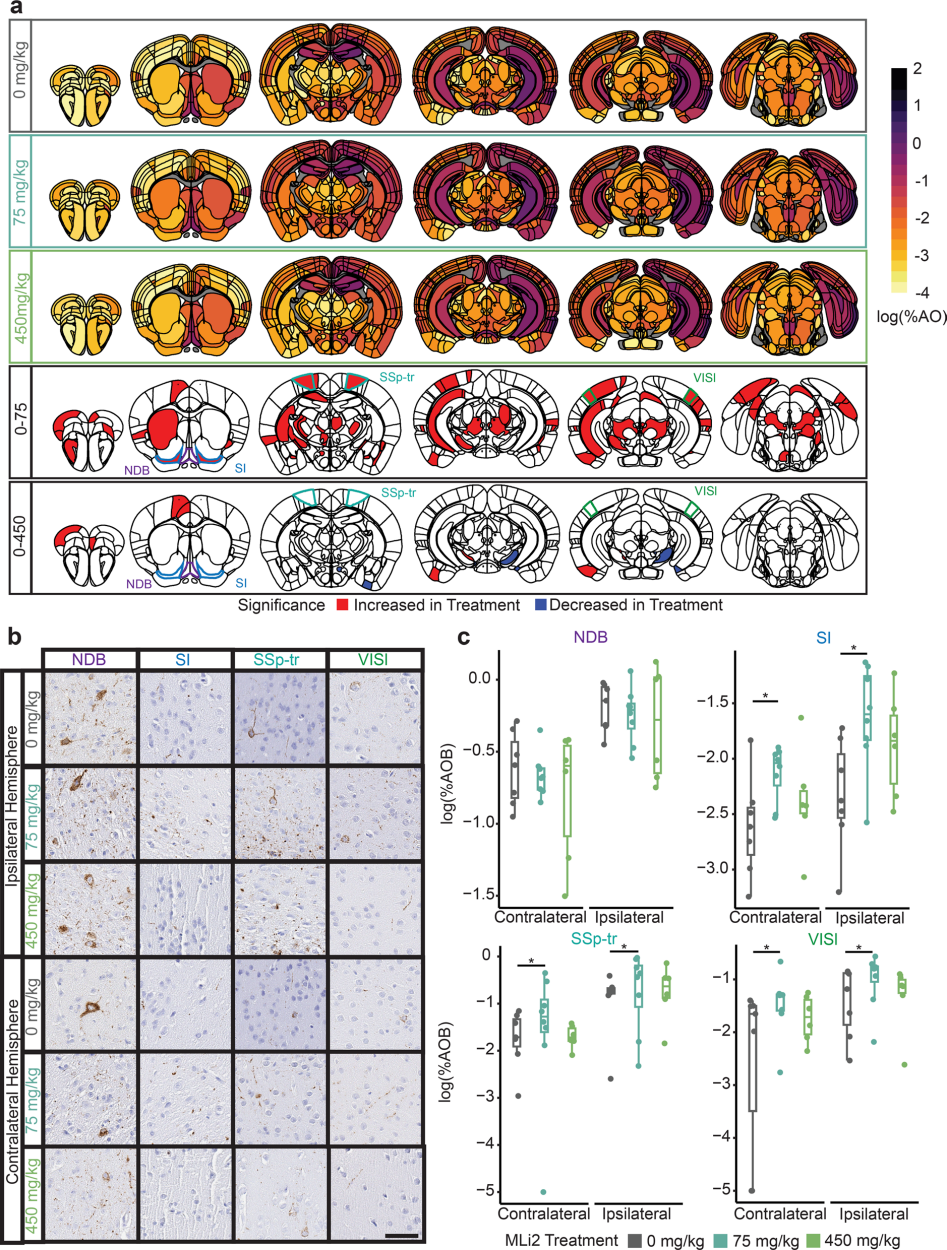
LRRK2 kinase inhibition shows no protective effect in wild-type mice. **a** Anatomic heatmaps of the mean regional tau pathology shown as log (% area occupied) at 6 MPI and second-generation *P*-values of regional statistical significance, calculated using ranged robust linear regression, of WT mice with 0 mg/kg MLi-2 compared to wild-type mice treated with 75 or 450 mg/kg MLi-2 (δ*P* = 0). Tau pathology was not quantified in white matter regions, so they are plotted as gray. Analyzed regions shown below are outlined. NDB: diagonal band nucleus; SI: substantia innominata; SSp-tr: primary somatosensory area, trunk; VISl: lateral visual area. **b** Representative images of selected regional tau pathology. Scale bar, 50 μm.** c** Quantification of selected brain regions. Regional tau shown as log (% area occupied). ns = not significant (δ*P* ≠ 0), *: Significant (δ*P* = 0). *N* = 7 (0 mg/kg MLi-2), 8 (75 mg/kg MLi-2), or 6 (450 mg/kg MLi-2)

If LRRK2 kinase activity is implicated in cases lacking a *LRRK2* mutation, then LRRK2 kinase inhibition may have a beneficial effect on pathology in wild-type mice. To test this hypothesis, we next compared wild-type mice treated with LRRK2 kinase inhibitors to controls. Again, we found minimal differences in tau pathology at 3 MPI (Additional file 1: Fig. S7). However, at 6 MPI, we observed a broad elevation in tau pathology in the 75 mg/kg dose group (Fig. 4a, Additional file 1: Fig. S8). Histologically, this elevation appeared to be primarily driven by neuritic pathology in those regions (Fig. 4b). Remarkably, this effect was nearly absent in the 450 mg/kg dose group. At either time point, these data do not support a protective role for LRRK2 kinase inhibitors in wild-type mice with tau pathology.

### LRRK2 kinase inhibition reduces cortical pathology in LRRK2^G2019S^ mice

**Fig. 5:**
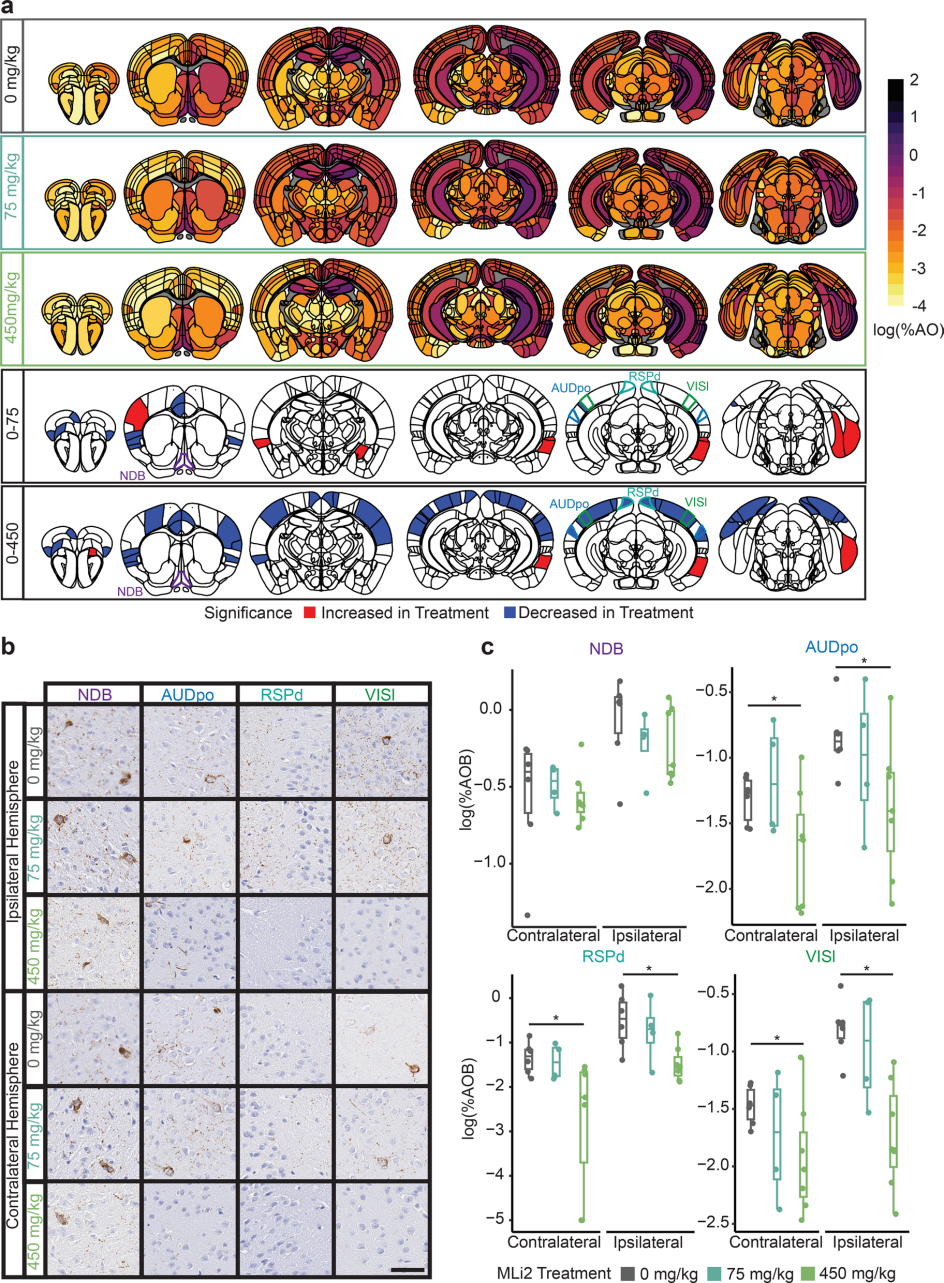
LRRK2 kinase inhibition reduces cortical pathology in LRRK2^G2019S^ mice. **a** Anatomic heatmaps of the mean regional tau pathology shown as log (% area occupied) at 6 MPI and second-generation *P*-values, calculated using ranked robust linear regression, of regional statistical significance of G2019S mice with 0 mg/kg MLi-2 compared to G2019S mice treated with 75 or 450 mg/kg MLi-2 (δ*P* = 0). Tau pathology was not quantified in white matter regions, so they are plotted as gray. Analyzed regions shown below are outlined. NDB: diagonal band nucleus; AUDpo: posterior auditory area; RSPd: retrosplenial area, dorsal part; VISl: lateral visual area. **b** Representative images of selected regional tau pathology. Scale bar = 50 μm.** c** Quantification of selected brain regions. Regional tau shown as log (% area occupied). ns = not significant (δ*P* ≠ 0), *: significant (δ*P* = 0). *N* = 6 (0 mg/kg MLi-2), 4 (75 mg/kg MLi-2), or 7 (450 mg/kg MLi-2)

LRRK2^G2019S^ and related mutations have been demonstrated to elevate LRRK2 kinase activity. Therefore, we next assessed the ability of LRRK2 kinase inhibitor MLi-2 to alter tau pathology progression in LRRK2^G2019S^ knock-in mice. Similar to the genotype effect, we observed minimal treatment effect at 3 MPI (Additional file 1: Fig. S7), suggesting that kinase activity is not essential for initial tau seeding. However, at 6 MPI, there was a broad decrease in cortical tau pathology in mice treated with 450 mg/kg MLi-2, but not 75 mg/kg MLi-2 (Fig. 5a, b). The main exception to this pattern was the entorhinal cortex, which is highly connected to the injection site. It is interesting that full inhibition of LRRK2 kinase activity seems to be required for this effect, despite the fact that 75 mg/kg MLi-2 should have reduced LRRK2 kinase activity below wild-type levels. The increase of tau pathology in the entorhinal area and decrease in other cortical regions suggests that the primary pathology seeding is not affected by LRRK2 kinase activity, unlike the progression to other brain regions. However, these broad changes can be difficult to assess region-by-region, so we turned to computational modeling of progression.

### LRRK2^G019S^ and LRRK2 kinase inhibition impact network progression of tau pathology

Previous work using computational modeling of intracellular pathology progression has demonstrated that anatomical connectivity is a major constraint on pathology progression [16, 18, 22]. While this type of modeling is useful for understanding how pathology progresses through the brain and the factors underlying regional vulnerability, we hypothesized that it could also be useful for understanding the impact of compound administration on network dynamics of pathology spread. We therefore implemented linear diffusion modeling on the brain-wide tau pathology data collected in this study.

**Fig. 6:**
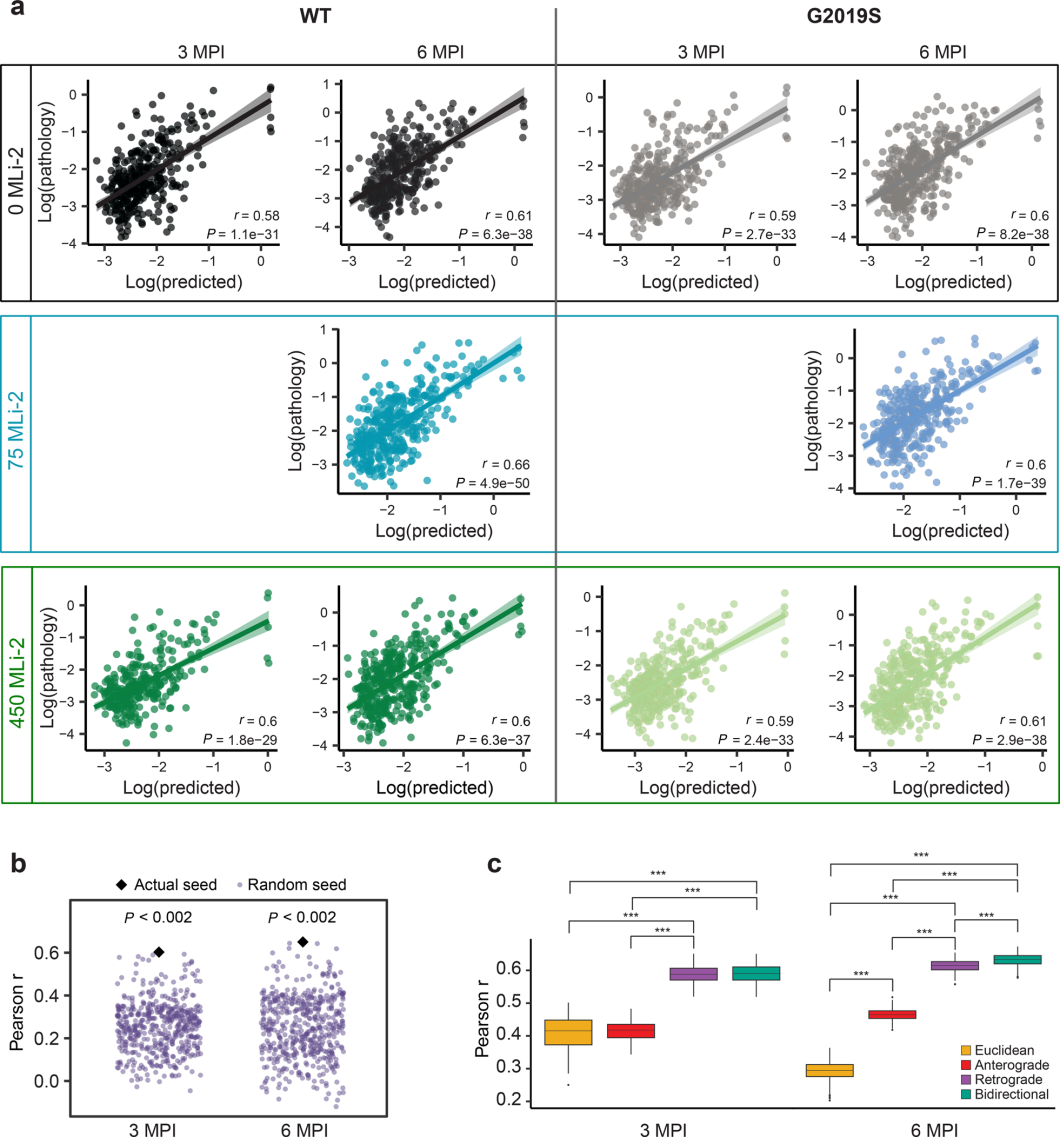
Tau pathology spread modeling. **a** Predictions of log tau pathology from linear diffusion models based on bidirectional (anterograde and retrograde) anatomical connections. Solid lines represent the line of best fit, and shading represents 95% confidence intervals. Each dot represents a different brain region where tau pathology was measured. **b** Comparison of Pearson’s *r* values obtained by fitting bidirectional spread models using actual (black diamond) and alternate (purple points) seed regions. Using the true seeds yielded a better fit than using random seeds at both 3 and 6 MPI. **c** Comparison of Euclidean, anterograde, retrograde, and bidirectional model fits across 500 held-out samples derived from 500 iterations of a cross-validation process. Retrograde and bidirectional spread models performed significantly better than the anterograde and Euclidean spread models at 3 MPI, and the bidirectional model performed significantly better than all other models at 6 MPI (***P < 0.002)

Since this was the first collection of high-resolution pathology data (844 regions versus our 134 regions previously [18]), we first confirmed that a linear diffusion model with anatomical connectivity as an input layer would be able to predict pathology in each of the genotypes and treatment groups. Notably, only 420 of the 844 regions we measured had corresponding connectivity data, largely due to the lack of cortical laminar connectivity data. Consistent with our previous study, we determined that our bidirectional spread model strongly predicted levels of tau pathology at 3 and 6 MPI in both WT and LRRK2^G2019S^ mice in all treatment groups (Fig. 6a, Additional file 1: Fig. S9). The overall model fit appeared to be unaffected by treatment with MLi-2. We validated the specificity of our model by comparing fits obtained with the actual seed regions to those obtained by randomly selecting 500 alternate sets of 5 seed regions. We found that using the true seed regions yielded a significantly better fit than using random seeds (*P* < 0.002; Fig. 6b), indicating that the model’s performance was specific to the experimental seed sites. Finally, we compared model fits obtained from our bidirectional spread model to those obtained by modeling spread only in the anterograde direction, only in the retrograde direction, or based on Euclidean distance. The bidirectional spread model yielded a significantly better fit than models based on anterograde spread or Euclidean distance at 3 MPI, and its performance was superior to anterograde, retrograde, and distance-based models at 6 MPI (*P* < 0.002).

**Fig. 7:**
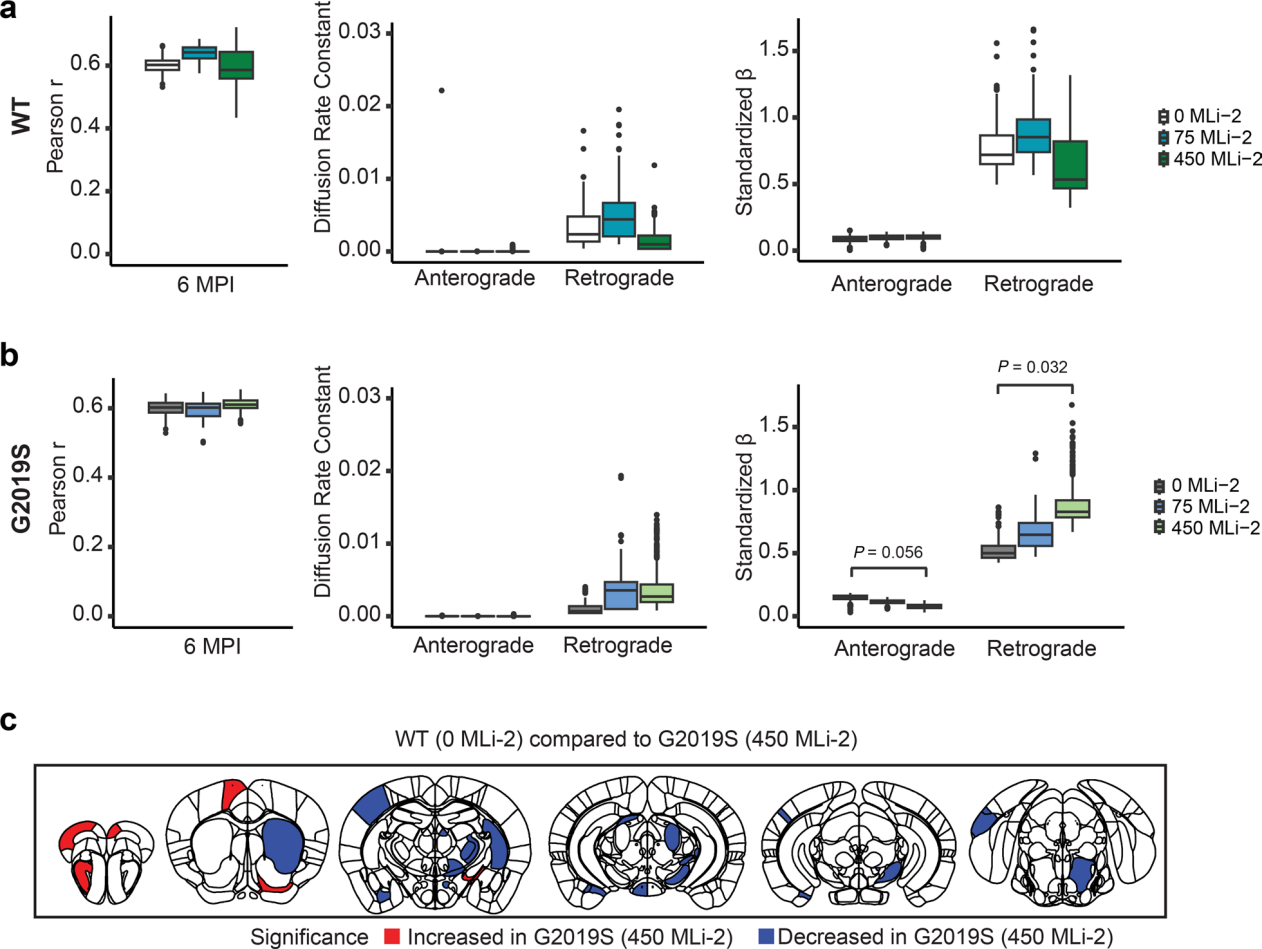
LRRK2^G019S^ and LRRK2 kinase inhibition impact network progression of tau pathology. Comparisons of Pearson* r*, diffusion rate constant and standardized β in WT mice (**a**) treated with 0, 75, or 450 mg/kg MLi-2 and LRRK2^G2019S^ mice (**b**) treated with 0, 75 or 450 mg/kg MLi-2. Distributions were generated for each group by obtaining 500 bootstrapped resamples and fitting the bidirectional diffusion model for each resample. Since data for the 75 mg/kg dose of MLi-2 were only available at 6 MPI, bootstrapping for each group was performed using only data from this time point. **c** Results of ranked robust linear regression analysis of regional tau pathology in WT mice under control treatment (0 mg/kg MLi-2) compared to LRRK2^G2019S^ mice treated with 450 mg/kg MLi-2. Blue and red colored regions have second-generation *P*-values equal to 0

To assess the impact of MLi-2 treatment on mechanisms of pathology spread, we fit the bidirectional spread model on bootstrapped samples of data from mice in each treatment group to obtain distributions of Pearson’s *r*, the diffusion rate constant, and standardized regression weights (Fig. 7a, b). We observed no difference in the overall fit of the bidirectional diffusion model across genotypes and treatment groups and no difference in the diffusion rate constant. LRRK2 kinase inhibition in wild-type mice had subtle effects on the standardized β weights that were not significant. However, the retrograde β weights were lower in LRRK2^G2019S^ mice, and MLi-2 treatment elevated those weights to levels more comparable to wild-type mice.

**Fig. 8:**
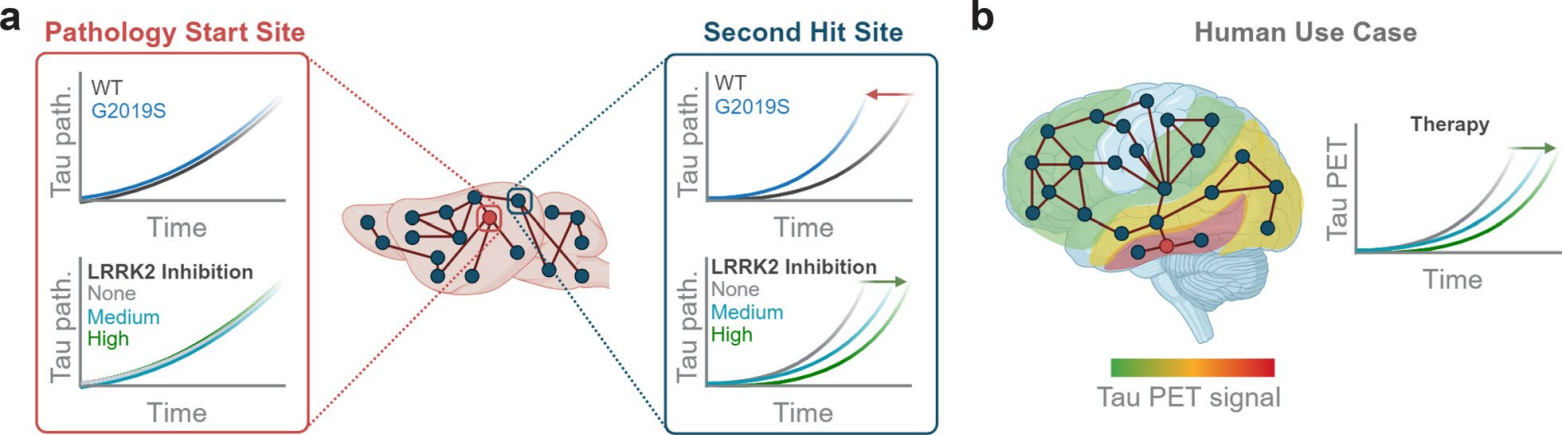
Model of LRRK2 impact on pathology progression. **a** Graphical summary of major study findings. Near the pathology start site and highly connected regions, there was minimal effect of either LRRK2^G2019S^ expression or LRRK2 kinase inhibition. However, in other regions, primarily cortical regions, there was an acceleration of tau pathology in LRRK2^G2019S^ mice that was reversed with LRRK2 kinase inhibition. **b** Proposed use of regional measurements and progression modeling to assess the impact of therapies either preclinically or clinically. Tau pathology can be assessed in living patients by PET imaging. Further, tau PET signal progresses in a predictable fashion, with individual variability. We propose that rather than assessing single regions, it may be more valuable to sample broadly and model the impact of therapies on progression

The modeling results support a shift in progression parameters induced in LRRK2^G2019S^ animals that is reversed by LRRK2 kinase inhibition. To assess this shift in individual regions, we compared wild-type mice on control diet to LRRK2^G2019S^ mice treated with 450 mg/kg MLi-2 for 6 months (Fig. 7c). The tau pathology was almost completely normalized by MLi-2 treatment, with almost all regions showing either no difference from wild-type mice or a significant reduction in pathology. Together, our data support a role for LRRK2 kinase activity in the progression of tau pathology and show that these effects can be reversed by chronic LRRK2 kinase inhibition, demonstrating the value of broad pathology evaluation and computational modeling in the evaluation of therapeutics (Fig. 8).

However, it is unclear how LRRK2^G2019S^ changes tau pathology progression. In addition to cell-autonomous explanations, there is growing evidence of the involvement of microglia in LRRK2-related phenotypes [41] and in tau pathology progression and neurodegeneration [42]. LRRK2 is present in microglia and upregulated in response to inflammatory stimuli [43]. Microglial morphology and inflammatory phenotypes have been reported to be similar between wild-type and LRRK2^G2019S^ mice, but upon stimulation, the LRRK2^G2019S^ microglia have increased reactivity [44, 45]. A recent study showed that LRRK2 inhibits microglial lysosomal degradative activity through transcriptional regulation of lysosomal gene expression (46). We therefore sought to determine whether there were histological changes in microglia in wild-type or LRRK2^G2019S^ mice treated with LRRK2 kinase inhibitor. We characterized visual cortex at 6 MPI where the tau pathology was increased in LRRK2^G2019S^ mice (Fig. 3) and reversed with LRRK2 kinase inhibition (Fig. 5). As a positive control, archival tissue from mice injected with the glucocerebrosidase inhibitor conduritol-β-epoxide (CBE) was included. We have previously demonstrated a robust cortical gliosis in these mice [22].

Mice were stained for GFAP, microglial marker Iba-1, and pS202/T205 tau. In contrast to the CBE-injected mice which had enlarged microglial cell bodies and increased GFAP signal, there was no dramatic differences in GFAP or Iba-1 signal in wild-type or LRRK2^G2019S^ mice (Additional file 1: Fig. S10a). The fluorescence intensity and average size of microglial cell bodies were determined through cell detection with intensity thresholding and object classification within QuPath to remove cell processes. Overall, wild-type and LRRK2^G2019S^ microglia had similar cell body size and Iba-1 intensity, with a slight trend to lower cell body size and higher Iba-1 intensity in the 450 mg/kg MLi-2-treated animals (Additional file 1: Fig. S10b, c). As a comparator, the Iba-1 intensity was elevated to approximately two folds in CBE-injected mice (Additional file 1: Fig. S10c). These experiments do not provide strong support of a gliosis phenotype in the mice studied here, but there may be more subtle changes in gliosis missed by this experiment.

## Discussion

LRRK2 mutations are the most common cause of familial PD, and the development of LRRK2 kinase inhibitors has been a major focus in PD research for the past 15 years [47]. Yet, the field has been slowed by two major roadblocks—concerns over the safety of LRRK2 kinase inhibition, and the lack of a validated biological readout for compound efficacy. Several studies have sought to understand the safety of LRRK2 inhibition [48], including recent Phase I clinical trials that assessed multiple doses in humans [49]. These safety studies concluded that while there are reproducible histological phenotypes associated with LRRK2 kinase inhibition, it seems possible to wash out these effects, and there is no overt change in lung function in animal studies or humans. However, the role of LRRK2 in PD pathophysiology has remained elusive, making testing of compounds on disease measures difficult. In the current study, we attempted to assess the impact of LRRK2^G2019S^ and LRRK2 kinase inhibition on tau pathology progression. We found that a biological effect was only observable when considering a large number of brain regions at extended time points, suggesting that it may be beneficial to expand compound efficacy studies outside of individual brain regions or time points.

We have previously demonstrated an effect of LRRK2^G2019S^ overexpression on the progression of tau pathology in AD PHF-injected mice [18]. Notably, the effects on pathology were only apparent at later time points. This absence of early change is consistent with a recent study that injected α-synuclein PFFs in the hippocampus of LRRK2 knockout and LRRK^G2019S^ knock-in mice, and observed no differences in α-synuclein pathology at 1 MPI [50]. Importantly, we and others often measure pathology in the injected region or regions with a high number of projections to the injected region. However, in the current and other studies [18, 19], these regions show minimal changes in pathology. Together, these data suggest that LRRK2 may enhance tau progression to distal sites, while reducing pathology in primary hit sites (entorhinal cortex). The cellular mechanism for this network-level phenomenon remains unclear but could be related to increased synaptic vesicle release [51] or altered autophagosome trafficking [52] that has been observed in LRRK2^G2019S^ neurons. If, for example, LRRK2 kinase activity leads to increased release of pathogenic tau, it may be expected to decrease pathology in certain hub nuclei like the entorhinal cortex but increase pathology in other recipient neurons. By the same logic, LRRK2 inhibition may increase pathology in the same hub nucleus (entorhinal cortex), while decreasing pathology elsewhere.

Alternatively, cell non-autonomous mechanisms could be responsible. LRRK2 is present in microglia and recent work has shown that inflammatory stimuli can upregulate LRRK2, which in turn may drive lysosomal dysfunction in microglia [43–46]. Microglia also play a prominent role in tauopathies. Work in transgenic tauopathy mouse models has shown that microglia drive tau pathology and tau-mediated neurodegeneration [53] and that tau pathology drives an activated microglial response [54] through cyclic GMP-AMP synthase and interferon signaling [55]. While we did not observe any dramatic shifts in microglial morphology in our study, it is possible that more subtle shifts in microglial homeostasis are responsible for the observed phenotypes, especially in the context of the substantial evidence relating LRRK2, microglia, and tau pathology.

Importantly, LRRK2 kinase inhibition was able to reverse the pathological changes in LRRK2^G2019S^ mice but showed no beneficial effect in wild-type mice. This finding is consistent with several previous studies that found no effect of LRRK2 kinase inhibition on pathology in wild-type mice or neurons [40, 56]. This data could be consistent with a role for microglia, where microglial activity is not dependent on LRRK2 kinase activity, but the presence of a LRRK2^G2019S^ mutation causes a perturbation in microglial homeostasis that is reversed with LRRK2 kinase inhibition. What was more surprising in the current study is that the 75 mg/kg MLi-2-treated wild-type mice showed elevations in tau pathology in several regions, while the 450 mg/kg MLi-2-treated wild-type mice were similar to controls. It is possible that in the absence of a LRRK2 mutation, LRRK2 kinase inhibition interferes with the basal function of LRRK2. However, the lack of a dose response in these mice suggests that this effect is not a direct correlate of LRRK2 kinase activity. While preclinical studies should be extrapolated with caution, our study suggests that LRRK2 kinase inhibitors may be beneficial in LRRK2^G2019S^ carriers, but not in non-carriers. However, there are many important differences between human PD and mouse models that could yield different outcomes in patients than in mice.

In addition to the broad pathology assessment, we also employed computational modeling of pathology progression to understand the impact of LRRK2^G2019S^ and kinase inhibition in our animal model. We implemented an improved pathology quantitation workflow to increase brain coverage to 844 regions. Given the novelty of this approach, we first wanted to validate its ability to predict pathology progression based on anatomical connectivity. We show a similar ability as our previous studies to predict pathology patterns, but with improved statistical confidence. Network modeling indicated that there were differences in standardized β weights, or the overall directionality of pathology progression, in LRRK2^G2019S^ mice that were reversed by LRRK2 kinase inhibition. Yet, this model was based only on the strength of inter-regional connections. Future models incorporating gene expression or functional activity may improve the predictivity [57] and thereby improve our understanding of the effects of gene mutations and therapies on pathology progression.

Together, broad assessments of pathology and network modeling can provide novel insights into disease progression and the impact of therapies [57]. In addition to their utility in preclinical models, we argue that they may prove useful in clinical evaluation of therapies, although further development in this area is required. Some therapies would be expected to act throughout the brain and would make broad assessments unnecessary. For example, anti-amyloid β immunotherapies can clear amyloid β plaques throughout the brain[58]. However, other therapies may reduce progression of pathology, while not impacting pathology in baseline regions. Tau pathology is measurable in living patients by positron emission tomography (PET) and may prove a valuable biomarker for disease progression [59, 60]. One can imagine a scenario in which a therapy does not change pathology at primary sites observed with a baseline PET, but subsequent PET imaging shows a reduction in non-primary regions compared to placebo. It is unclear if tau pathology is substantial enough in PD patients, or whether tau PET is sensitive enough to differentiate more subtle pathology changes, but this type of secondary analysis may be broadly applicable in neurodegenerative disease, if not in living patients, then possibly in post-mortem neuropathological studies.

There are several limitations to the current study. To make this study feasible within time constraints, we only measured two time points with a single LRRK2 kinase inhibitor. Longer time points may be necessary to provide a full picture of the impact of LRRK2 on pathology progression, and it would be important to show biological efficacy for compounds that are bound for the clinic. We also only used one variable, pS202/T205 tau (AT8), as a pathology measure in this study. The amount of time it takes to segment and register brain sections was prohibitive to assessing more than one measure, but improvements in this process may make this technique more streamlined in the future. We started LRRK2 kinase inhibition concurrent with tau pathology induction in this study, so the results could be perceived as preventative, not protective. However, given that minimal effects were seen at 3 MPI, we hypothesize that treatment could be delayed for some time with similar results.

## Conclusions

There are several advanced therapeutics targeting LRRK2 kinase activity in PD, yet our understanding of the role of LRRK2 in vivo is limited. The current study examined tau pathology progression in mice with and without a LRRK2 mutation treated with control or LRRK2 inhibitor diet. We found that the LRRK2^G2019S^ mutation accelerated tau pathology progression, which was reversed by LRRK2 kinase inhibition. We also established a novel workflow for the assessment of potential therapies on the network-level that we believe will be useful for future research.

### Supplementary Information

**Additional file 1**. **Table S1**: Characterization of PHF preparations from AD brains. **Figure S1**: Long-term MLi-2 impacts lung, but not the gross kidney morphology. **Figure S2**: Quantitative pathology workflow. **Figure S3**: Quantitative pathology analysis from all mice. **Figure S4**: Sex differences in tau pathology in wild-type mice. **Figure S5**: Representative staining from 3 MPI mice. **Figure S6**: Wild-type compared to LRRK2G2019S mice at 3 MPI. **Figure S7**: Tau pathology compared by treatment group at 3 MPI. **Figure S8**: Representative staining from 6 MPI mice. **Figure S9**: Examination of linear diffusion fits by hemisphere. **Figure S10**: Microglia quantification in caudal cortex of 6 MPI mice.

## Data Availability

The data that support the findings of this study together with the code used for data analysis are available here: https://github.com/jkbrynildsen/tau-spread. Primary code used to analyze data and generate linear diffusion models is available here: Initial conversion of Nutil files to matrix output and grid heatmap generation: https://github.com/DaniellaDeWeerd/NutilToUsable (RRID:SCR_024753). Brain heatmaps and differential analysis of area occupied: https://github.com/vari-bbc/Mouse_Brain_Heatmap. Computational models: https://github.com/jkbrynildsen/tau-spread.

## Abbreviations

LRRK2: Leucine-rich repeat kinase 2
PD: Parkinson’s disease
AD: Alzheimer’s disease
PHF: Paired helical filament
MPI: Months post-injection
WT: Wild-type
proSP-C: Prosurfactant protein C
PET: Positron emission tomography
SSp-m: Primary somatosensory area, mouth
SI: Substantia innominate
CBE: Conduritol-β-epoxide
