# Supplementary material for: LRRK2 kinase inhibition reverses G2019S mutation-dependent effects on tau pathology progression
Source: Transl Neurodegener. 2024 Mar 4;13:13. doi: 10.1186/s40035-024-00403-2 (PMC10910783; doi:10.1186/s40035-024-00403-2)
Supplement: Supplementary file 1 — Additional file 1. Table S1: Characterization of PHF preparations from AD brains. Figure S1: Long-term MLi-2 impacts lung, but not the gross kidney morphology. Figure S2: Quantitative pathology workflow. Figure S3: Quantitative pathology analysis from all mice. Figure S4: Sex differences in tau pathology in wild-type mice. Figure S5: Representative staining from 3 MPI mice. Figure S6: Wild-type compared to LRRK2G2019S mice at 3 MPI. Figure S7: Tau pathology compared by treatment group at 3 MPI. Figure S8: Representative staining from 6 MPI mice. Figure S9: Examination of linear diffusion fits by hemisphere. Figure S10: Microglia quantification in caudal cortex of 6 MPI mice. [file 40035_2024_403_MOESM1_ESM.docx]

**Additional file 1:**

**Supplemental Information for**

**LRRK2 kinase inhibition reverses G2019S mutation-dependent effects on tau pathology spread**

Noah Lubben^1,2^, Julia K. Brynildsen^2,3^, Connor M. Webb^4^, Howard L. Li^4^, Cheryl E. G. Leyns^5^, Lakshmi Changolkar^4^, Bin Zhang^4^, Emily S. Meymand^4^, Mia O’Reilly^3^, Zach Madaj^1^, Daniella DeWeerd^1,2^, Matthew J. Fell^5^, Virginia M.Y. Lee^4^, Dani S. Bassett^2,3,6-10^, Michael X. Henderson^1,2^*

| Case | Age | Sex | PMI (hours) | Total protein (μg/μL) | Tau (μg/μL) | Tau (% protein) | α-Syn (μg/mL) | Aβ 1-40 (ng/mL) | Aβ 1-42 (ng/mL) |
| --- | --- | --- | --- | --- | --- | --- | --- | --- | --- |
| AD Case 1 | 89 | F | 18 | 7.1 | 0.67 | 9.4 | 0.620 | 10.74 | 102.71 |
| AD Case 2 | 74 | M | 18 | 6.7 | 0.74 | 11.0 | 0.024 | 3.57 | 57.23 |
| AD Case 3 | 82 | F | 7 | 4.6 | 0.45 | 9.8 | 0.029 | 16.79 | 131.53 |

**Table S1. Characterization of PHF preparations from AD brains.** Three cases were extracted for this study. Information, including the post-mortem interval (PMI) for these cases is displayed in the table, in addition to protein concentrations used to evaluate the amount of protein to inject.


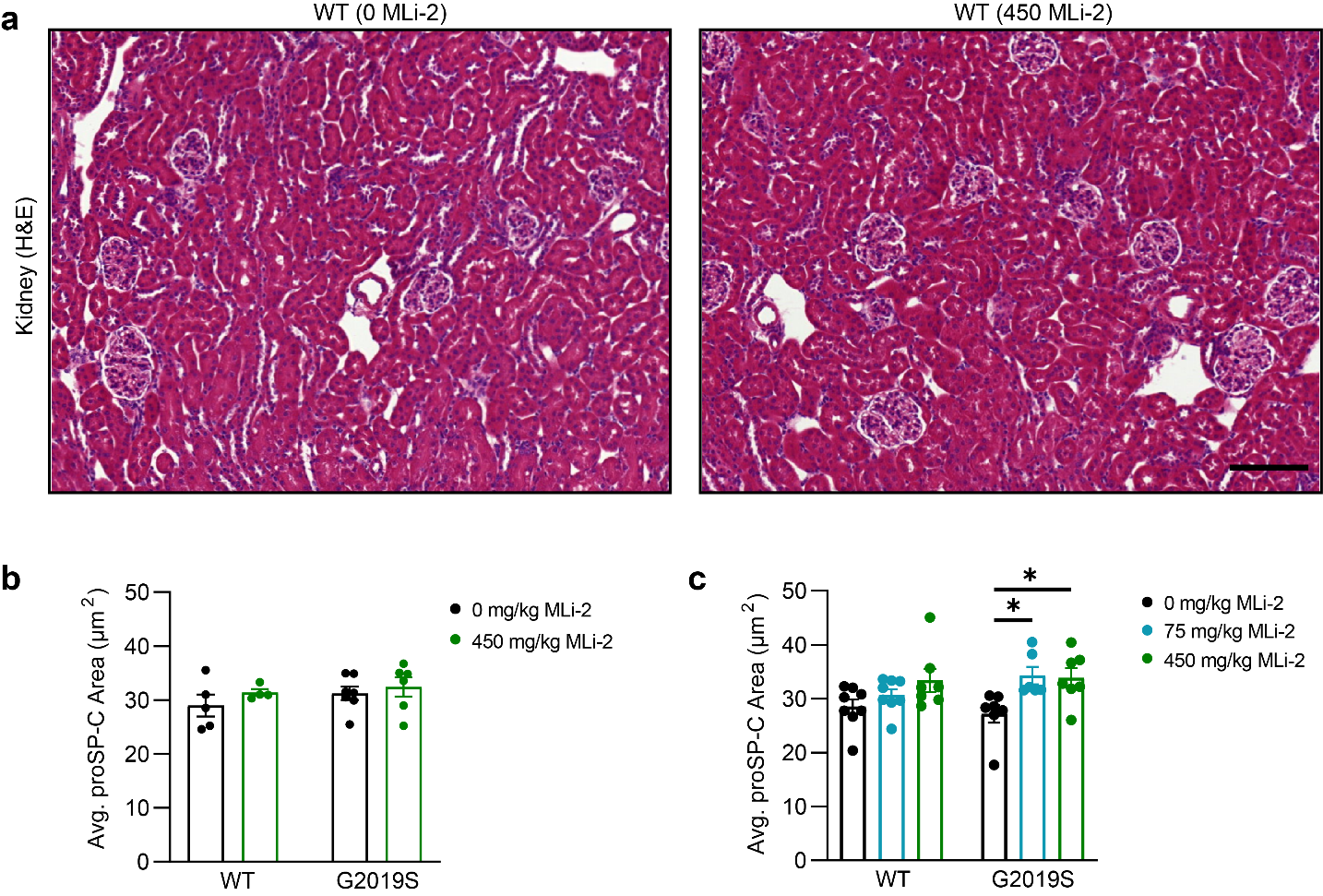


**Fig. S1 Long-term MLi-2 impact lung, but not gross kidney morphology.** **a** All experimental animals had their kidneys removed, sectioned, and stained by hematoxylin and eosin. No gross morphological differences were apparent in any of the cohorts. As an example, kidneys from wildtype mice treated with 0 mg/kg MLi-2 are compared above to wildtype mice treated with 450 mg/kg MLi-2 for 6 months. Scale bar = 100 µm. As an alternative method of quantification of lung phenotype, lung pro-SP-C measures are reported here as average areas per mouse for mice 3 MPI **b** or 6 MPI **c**. *p<0.05, 2-way ANOVA with Dunnett’s multiple comparisons test to compare MLi-2 doses.


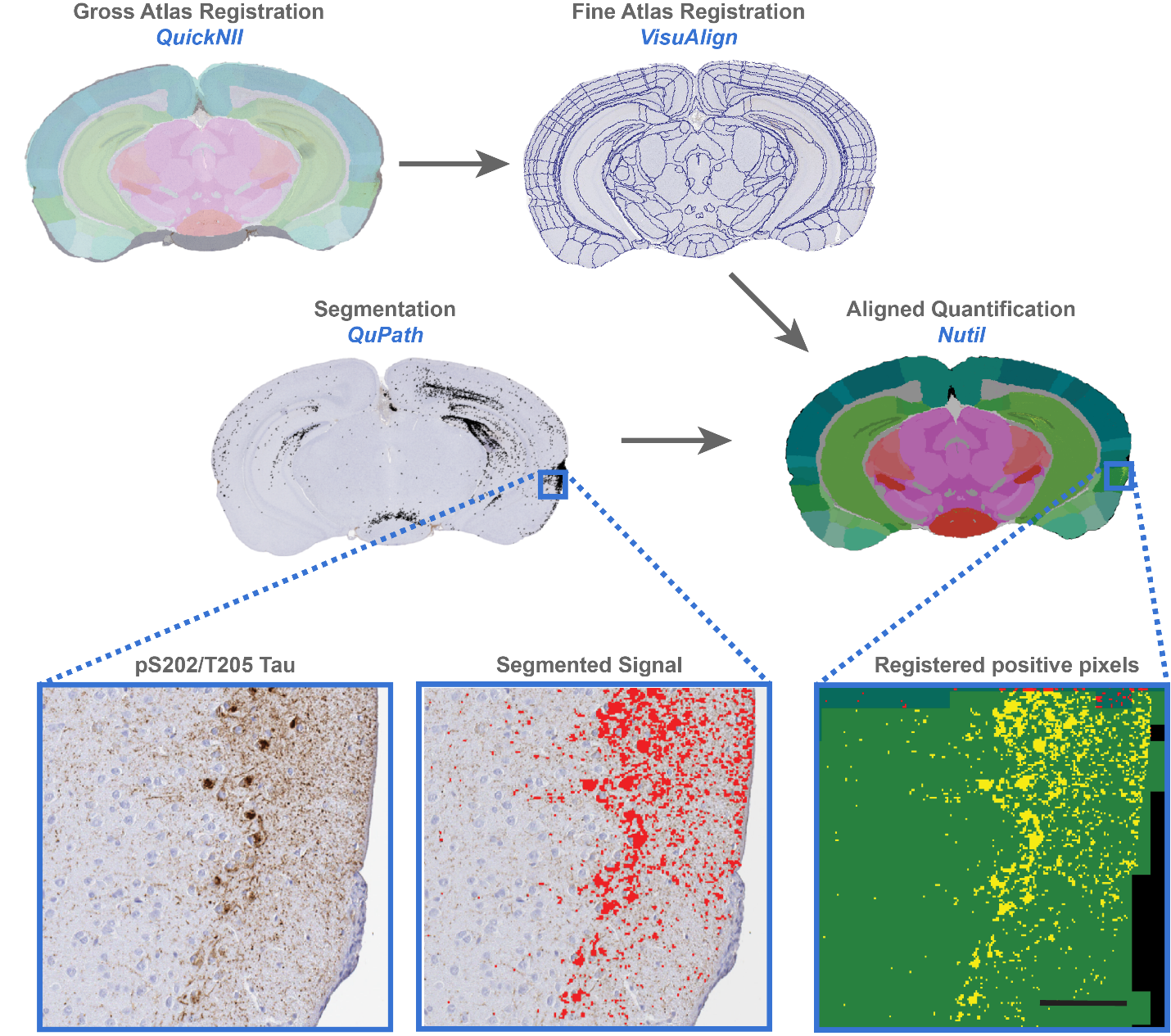


**Fig. S2 Quantitative pathology workflow.** Brains from mice injected with AD PHFs were mounted in paraffin blocks, sectioned and stained for pathological tau (AT8, pS202/T205). Stained tissue was then scanned into a digitized format. Segmentation and registration to the Allen Brain Atlas CCFv3 was performed with a modified version of the QUINT workflow. Pathology was segmented in QuPath based on pixel intensity. A parallel image was registered to the Allen Brain Atlas using QuickNII software for gross registration and VisuAlign for fine registration, including non-linear warp transformation. Finally, segmentation and registration data were merged in Nutil to provide outputs of percentage of areas occupied for each brain region. Representative pathology segmentation and registration are shown in enlarged images for the entorhinal region of this brain. Scale bar, 100 µm.


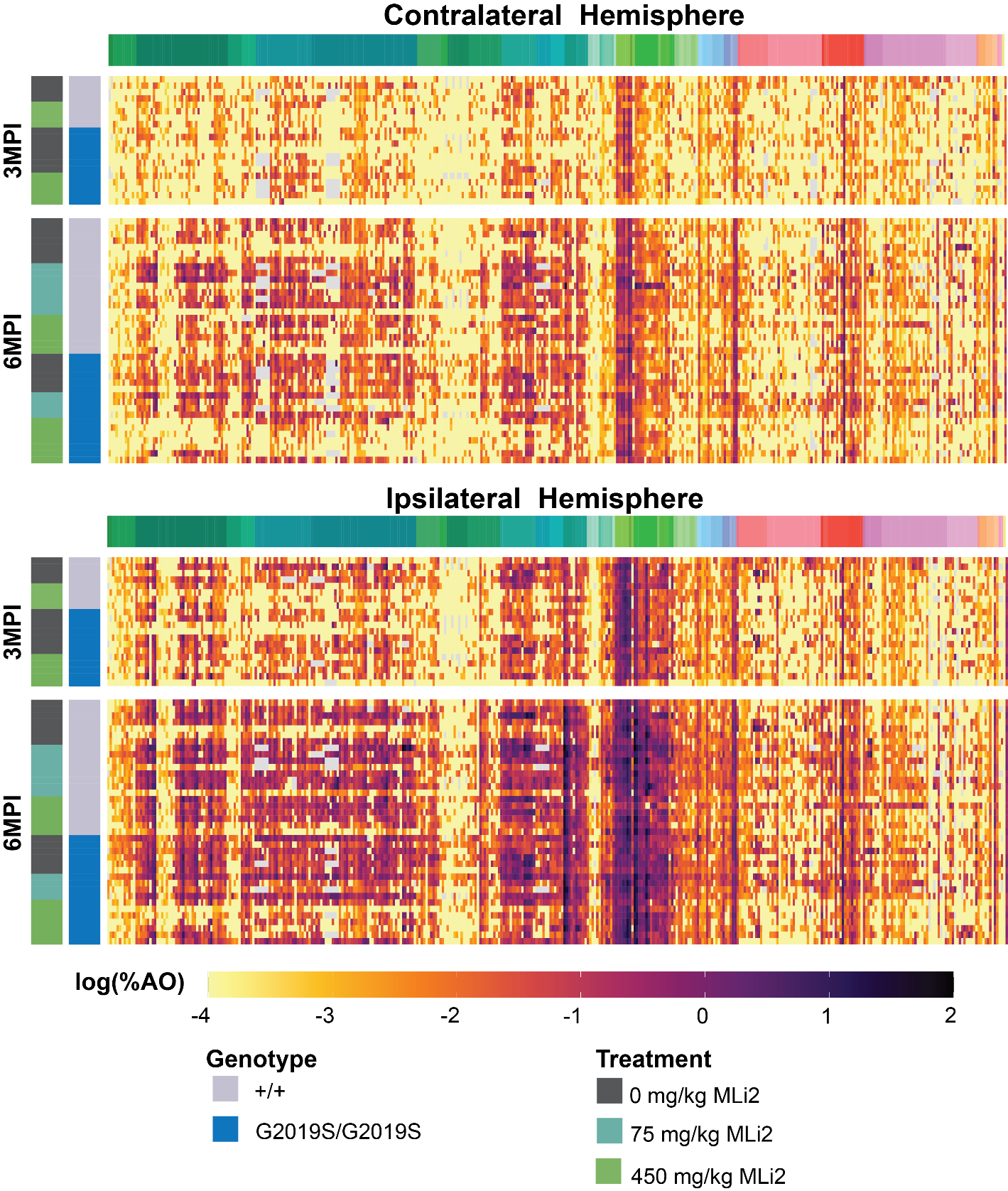


**Fig. S3 Quantitative pathology analysis from all mice.** Log percentage area occupied is plotted for all measured regions and mice, with the treatment and genotypes noted on the x-axis, and the brain region denoted on the y-axis by Allen Brain Atlas region coloration.


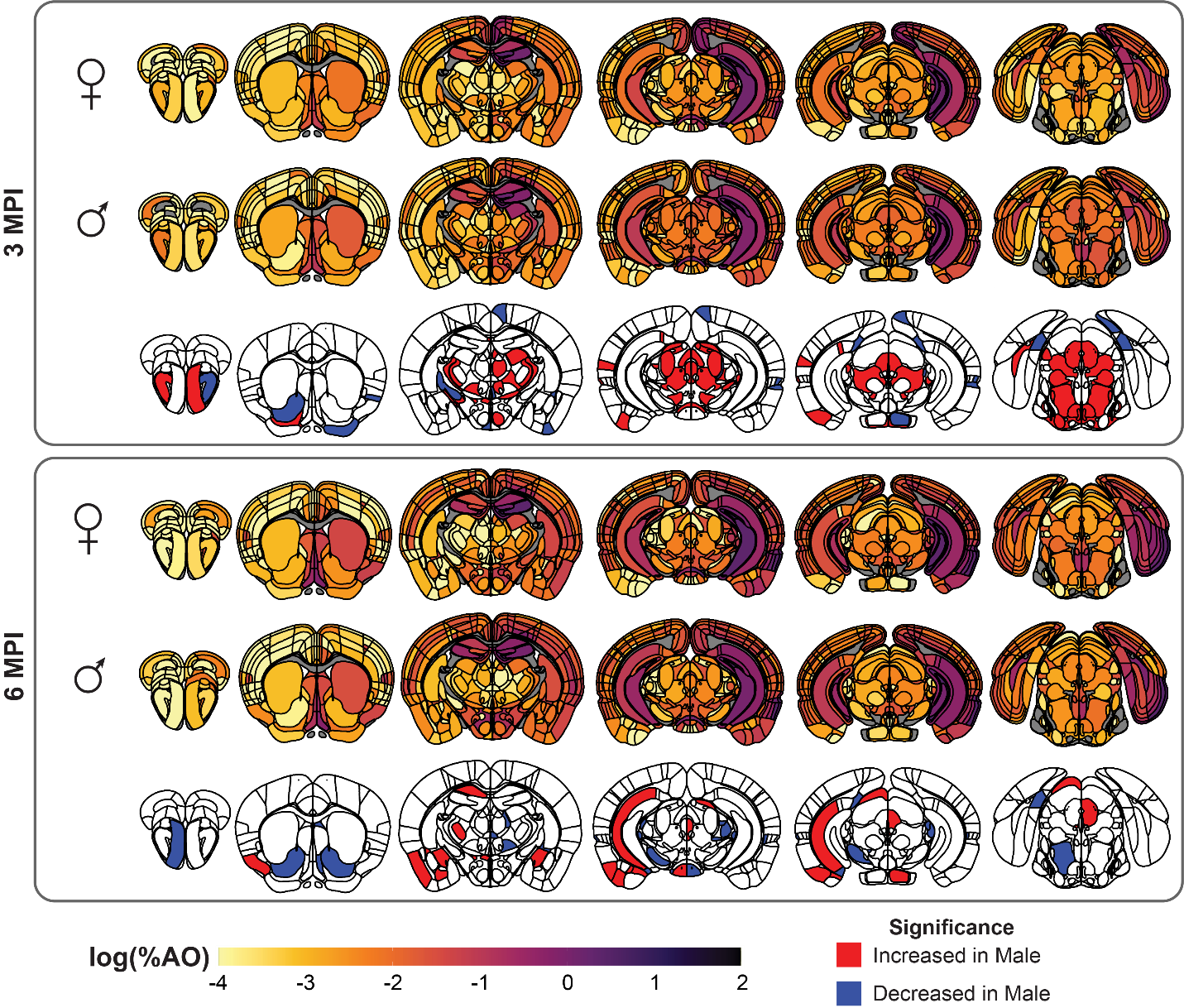


**Fig. S4 Sex differences in tau pathology in wildtype mice.** Anatomical heatmaps of average log percentage area occupied with pS202/T205 tau is plotted for male and female mice at 3 and 6 months post-injection and second-generation *p*-values of regional statistical significance of male compared to female mice (δp=0). While the majority of cortical regions show no different between males and females, several subcortical regions show differences. Most notable are the increase in thalamic and mesencephalic regions and CA1 region of the contralateral hippocampus in male mice.


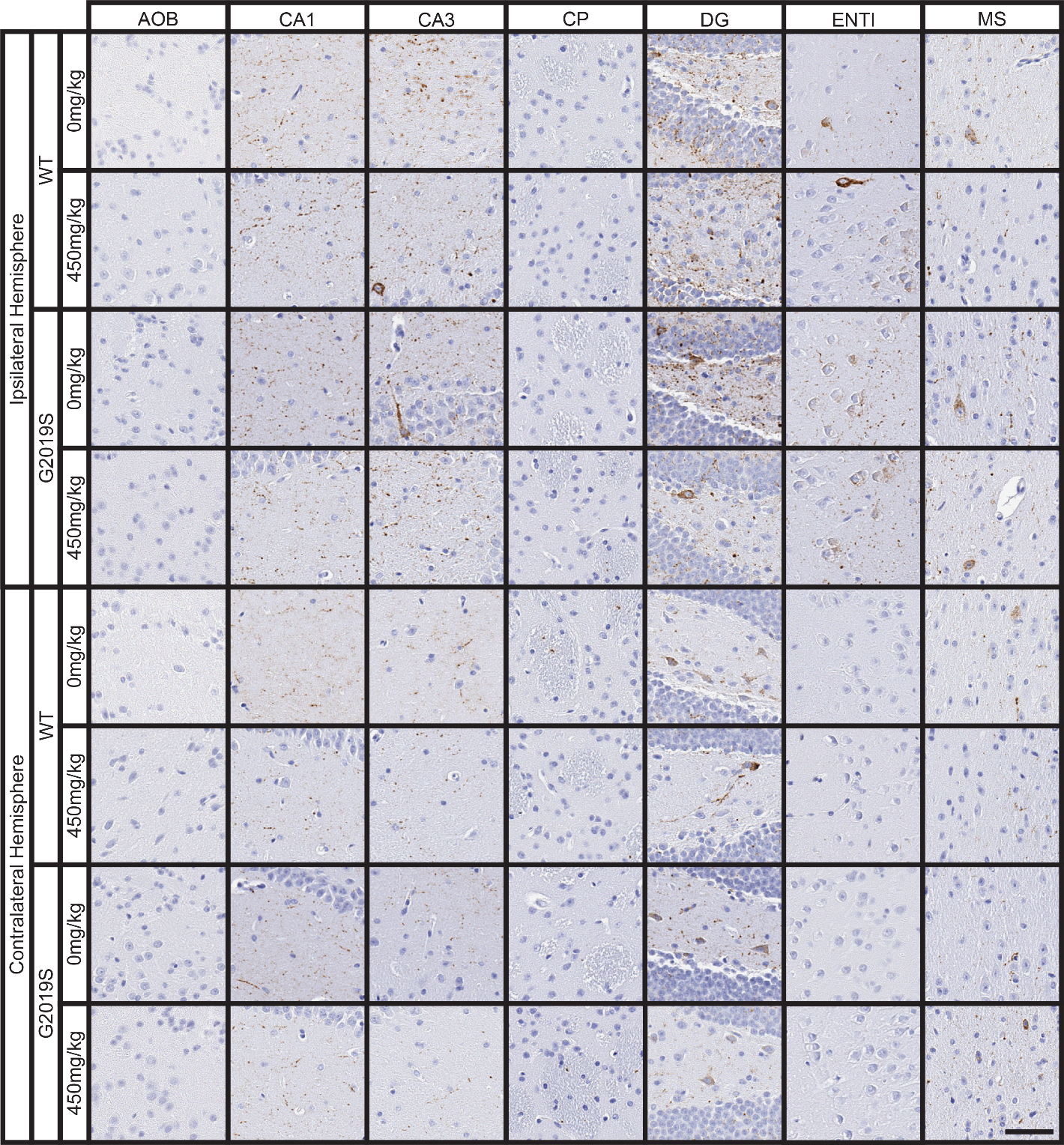


**Fig. S5 Representative staining from 3 MPI mice.** Representative images of selected regional tau pathology from 3 MPI mice. AOB: accessory olfactory bulb, CA1: field CA1 of hippocampus, CA3: field CA3 of hippocampus, CP: caudoputamen, DG: dentate gyrus, ENTl: entorhinal area, lateral part, MS: medial septal nucleus. Scale bar, 50 μm.

**
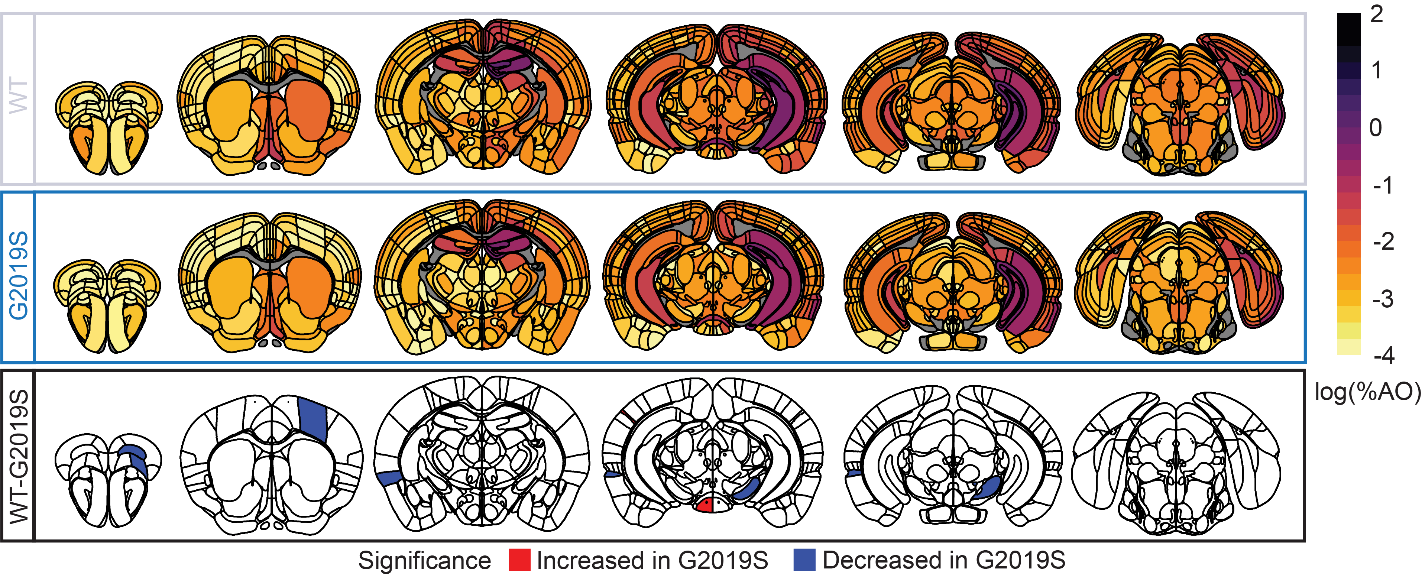
**

**Fig. S6 Wildtype compared to LRRK2^G2019S^ mice at 3 MPI.** Anatomic heatmaps of mean regional tau pathology shown as log (% area occupied) at 3 MPI and second-generation *p*-values of regional statistical significance of wildtype mice compared to G2019S mice (δp=0). Tau pathology was not quantified in white matter regions, so they are plotted as gray. There were minimal differences between genotypes at this timepoint.

**
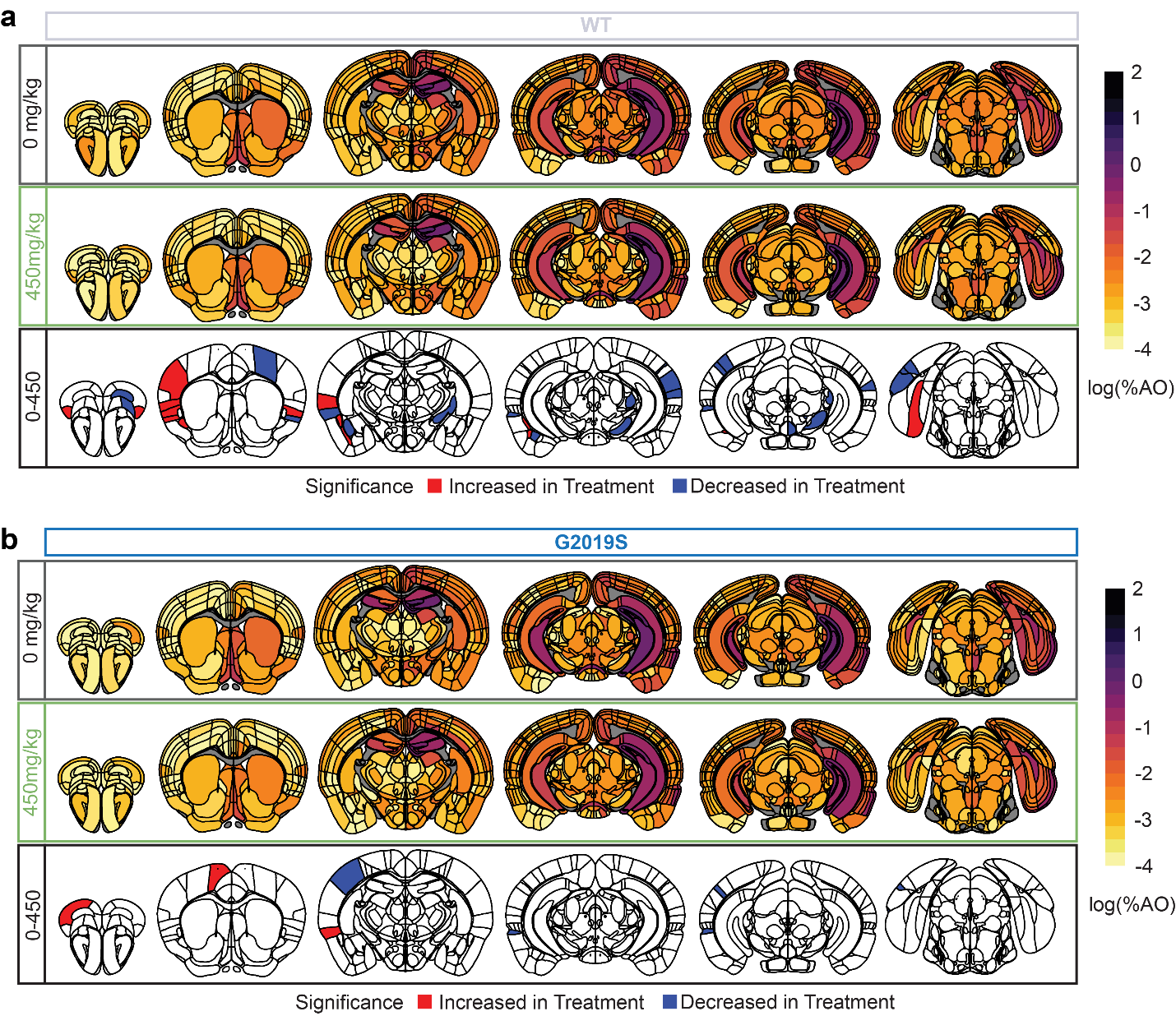
**

**Fig. S7 Tau pathology compared by treatment group at 3 MPI.** **a** Anatomic heatmaps of mean regional tau pathology shown as log (% area occupied) at 3 MPI and second-generation *p*-values of regional statistical significance of wildtype mice treated with 0 mg/kg MLi-2 compared to 450 mg/kg MLi-2 (δp=0). **b** Anatomic heatmaps of mean regional tau pathology shown as log (% area occupied) at 3 MPI and second-generation *p*-values of regional statistical significance of LRRK2^G2019S^ mice treated with 0 mg/kg MLi-2 compared to 450 mg/kg MLi-2 (δp=0). There were minimal differences associated with treatment at this timepoint.

**
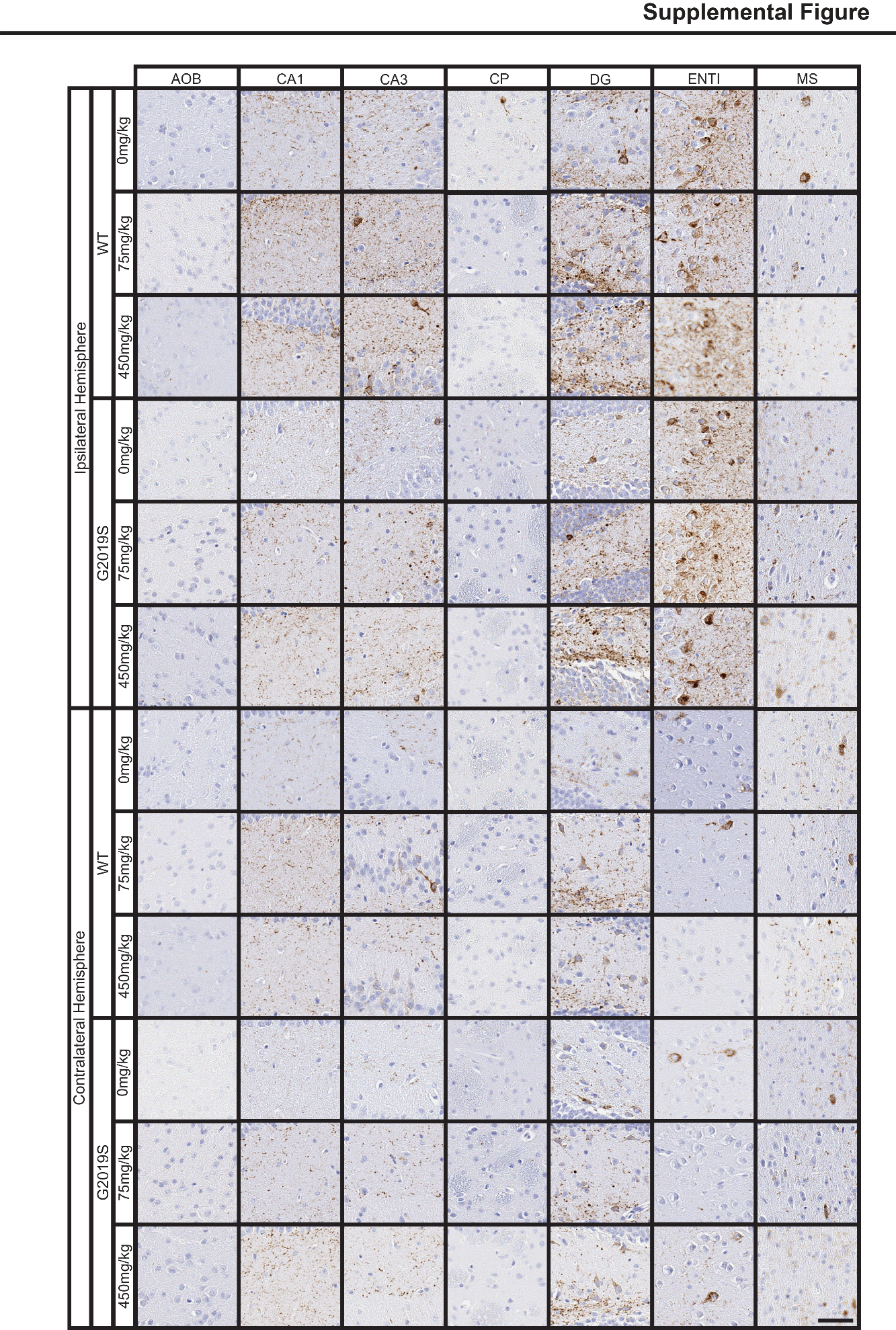
**

**Fig. S8 Representative staining from 6 MPI mice.** Representative images of selected regional tau pathology from 6 MPI mice. Scale bar, 50 μm.


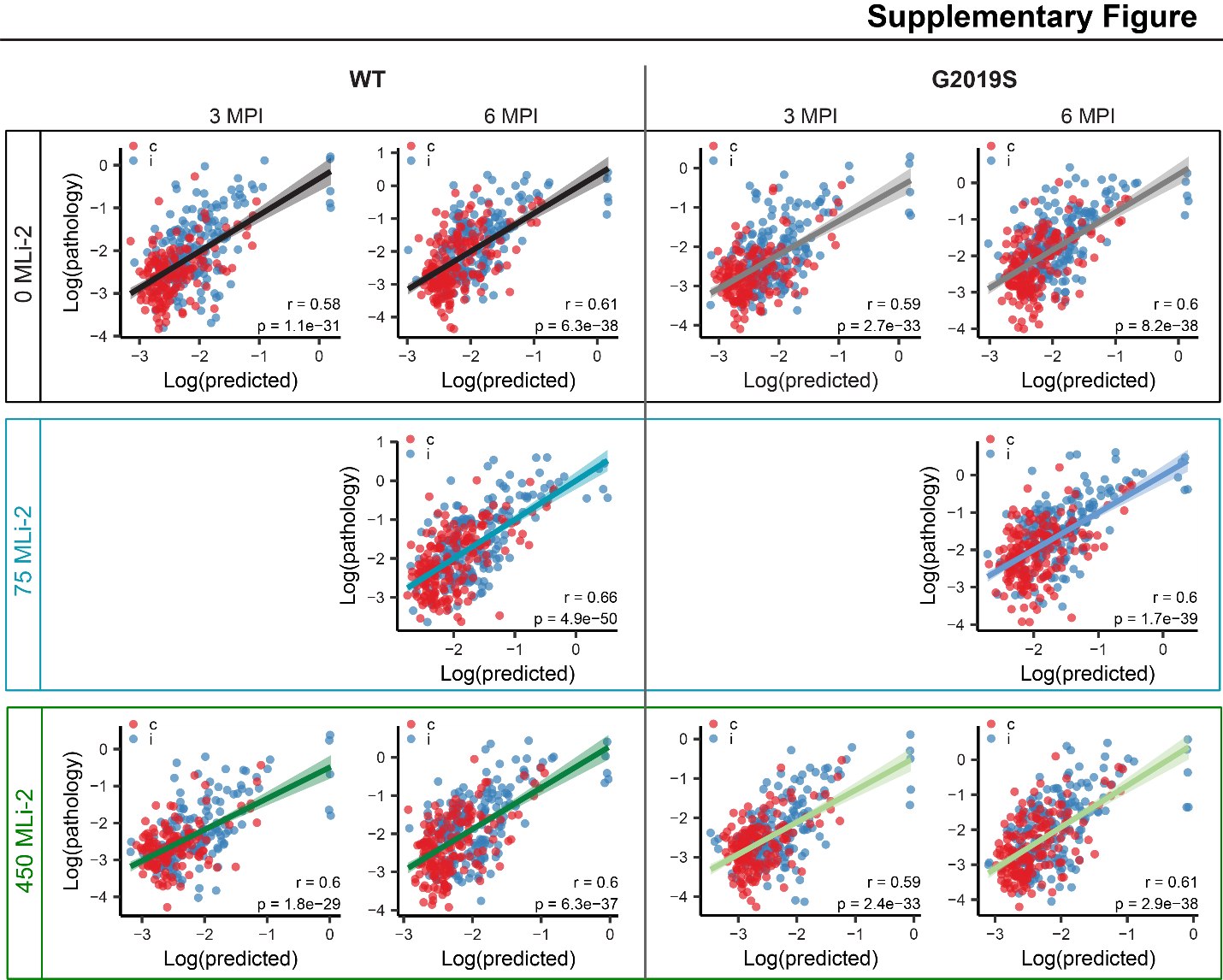


**Fig. S9 Examination of linear diffusion fits by hemisphere.** Predictions of log tau pathology from linear diffusion models based on bidirectional (anterograde and retrograde) anatomical connections. Solid lines represent the line of best fit, and shading represents 95% confidence intervals. This is the same data as plotted in **Fig. 6a**, except ipsilateral (blue) and contralateral (red) brain regions are colored differently.


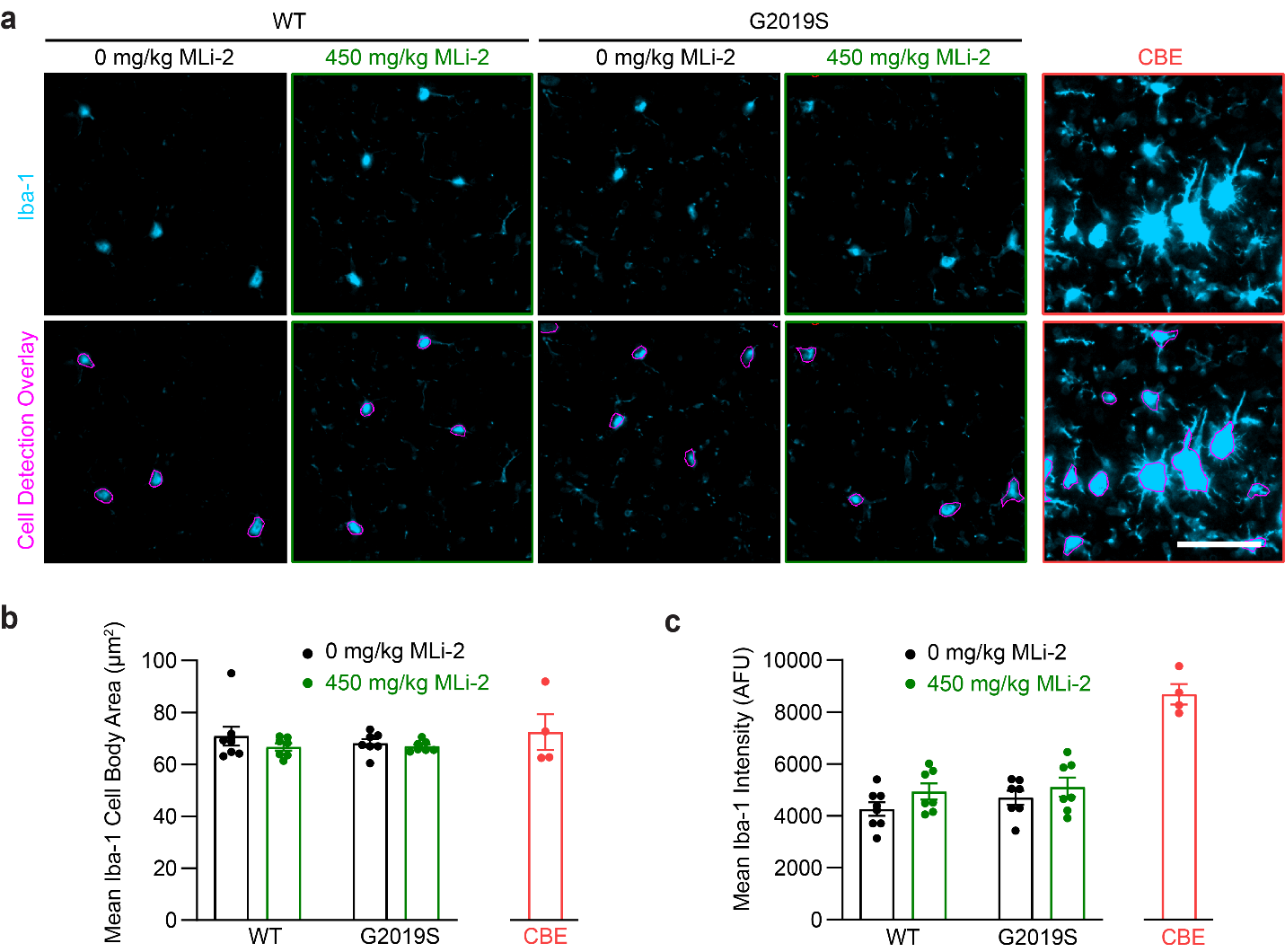


**Fig. S10 Microglia quantification in caudal cortex of 6 MPI mice. a** Images of Iba-1 staining in the visual cortex of wildtype and LRRK2^G2019S^ mice treated with 0 or 450 mg/kg MLi-2 in diet. Wildtype mice treated with CBE were included as a positive control for microgliosis. The tope panels include Iba-1 staining, while the bottom panels also include an overlay of the cell detection used for quantification. Scale bar, 50 µm. **b** Mean cell body area calculated based on cell detection. **c** Mean Iba-1 intensity in detected cell bodies in arbitrary fluorescence units (AFU). Genotype and MLi-2 treatment group were compared by 2-way ANOVA for both panels **b** and **c**. No significant effect was seen for genotype, treatment, or treatment within genotype by Sidak’s multiple comparisons test.
